# Contraception use and pregnancy in women receiving a 2-dose Ebola vaccine in Rwanda: A retrospective analysis of UMURINZI vaccination campaign data

**DOI:** 10.1371/journal.pmed.1004508

**Published:** 2025-02-11

**Authors:** Rosine Ingabire, Julien Nyombayire, Amelia Mazzei, Jean-Baptiste Mazarati, Jozef Noben, Michael Katwere, Rachel Parker, Sabin Nsanzimana, Kristin M. Wall, Tyronza Sharkey, Felix Sayinzoga, Amanda Tichacek, Niina Hammoud, Ellen Martinson, Ben Magod, Susan Allen, Etienne Karita

**Affiliations:** 1 Center for Family Health Research, Kigali, Rwanda; 2 Rwanda Biomedical Center, Kigali, Rwanda; 3 Janssen R&D, Beerse, Belgium; 4 Janssen Vaccines and Prevention, Leiden, the Netherlands; 5 Rwanda Zambia Health Research Group, Department of Pathology and Laboratory Medicine, School of Medicine, Emory University, Atlanta, Georgia, United States of America; 6 Rwanda Ministry of Health, Kigali, Rwanda; 7 Janssen Biologics Europe, Leiden, the Netherlands; Pasteur Network, FRANCE

## Abstract

**Background:**

Rwandan individuals bordering the Democratic Republic of the Congo (DRC) are at-risk of Ebola virus disease. A 2019 to 2021 vaccination campaign called UMURINZI offered a Janssen Vaccines & Prevention B.V. 2-dose heterologous Ebola vaccine regimen (Ad26.ZEBOV, MVA-BN-Filo) to Rwandan individuals aged ≥2 years and not pregnant. In this region with high rates of pregnancy, preventing pregnancy until their second dose of the Ebola vaccine is essential to ensure full protection. This analysis describes contraceptive use, pregnancy incidence, serious adverse events (SAE), and the effect of pregnancy and SAE on receipt of the second dose among women in the UMURINZI vaccination campaign.

**Methods and findings:**

During the vaccination campaign, women who were fertile and sexually active were counseled as part of the campaign by trained UMURINZI nursing staff about preventing pregnancy until dose two (56 days post-dose one) and offered contraception. Women were followed up to their second dose appointment. Contraception, pregnancy incidence, and SAE were recorded. Of the 47,585 fertile and sexually active women, the mean age was 28·0 years (standard deviation 9·9 years), 54·7% (*n* = 26,051) were from Rubavu and 45·3% (*n* = 21,534) were from Rusizi, and 71·9% (*n* = 34,158) had not crossed the DRC border in the last year. Sixty-six percent (66·6%, *n* = 31,675) were not using modern contraception at baseline and 19·1% (*n* = 9,082) were using hormonal implants, 10·9% (*n* = 5,204) injectables, 2·9% (*n* = 1,393) oral contraceptive pills (OCPs), and 0·5% (n = 231) intrauterine devices. After contraceptive counseling, 8·0% (*n* = 2,549) of non-users initiated a method of contraception and 3·6% (*n* = 50) of OCP users switched to a more effective method. Of the 969 incident pregnancies detected after dose one, 18·8% (*n* = 182) resulted in an obstetric SAE, primarily due to spontaneous abortion which occurred in 16·0% (*n* = 155) of all incident pregnancies. Other obstetric SAE included 14 blighted ova, 9 stillbirths, 1 termination due to hydrops fetalis, 1 cleft palate, and 2 threatened abortions resulting in normal deliveries. Six pregnant women had a non-obstetric SAE (0·6%), including 1 postpartum death from COVID-19 and 5 hospitalizations. Among the 74,002 women without an incident pregnancy detected after dose one, 0·01% (*n* = 4) had an SAE; 2 were fatalities due to hypertension and diabetes in one case and seizures in the other, and the other 2 were hospitalizations. No SAE were determined to be related to the vaccine by the program physicians. Among the 74,002 women without an incident pregnancy detected after dose one, 94·6% (*n* = 69,986) received dose two; in contrast, among the 969 women with an incident pregnancy detected after dose one, 34·5% (*n* = 334) received dose two after pregnancy completion.

**Conclusions:**

Many fertile and sexually active women who sought vaccination during UMURINZI were not using contraception prior to vaccination, and contraceptive method uptake after family planning counseling and method provision was low. Most women who became pregnant after the first vaccination dose did not receive the second dose, thus potentially reducing protection against Ebola. Family planning messaging for this context should be developed and pilot-tested. The estimated risk of spontaneous abortion was similar to previous estimates from Rwanda and other African countries.

## Author summary

**Why was this study done?:**

- In 2019, the UMURINZI Ebola vaccination campaign was launched in Rwanda as a result of repeated outbreaks across the border in the Democratic Republic of the Congo (DRC) and the high volume of Rwandan individuals crossing the border regularly.
- Pregnant women were excluded from UMURINZI due to limited vaccine safety data in pregnant women at the time.
- Unplanned incident pregnancy after vaccine dose one could reduce the number of women who receive dose two.
- To the best of our knowledge, there is no identifiable literature about offering family planning methods for short-term pregnancy prevention during multidose vaccination campaigns.

**What did the researchers do and find?:**

- We describe contraceptive use, pregnancy incidence, serious adverse events (SAE), and the associations between pregnancy and SAE and the outcome of receipt of the second vaccine dose among women in the campaign.
- Contraceptive method use was low and uptake after family planning counseling and method provision was also low.
- Many women who became pregnant after the first vaccine dose did not receive the second dose, thus potentially reducing protection against Ebola.

**What do these findings mean?:**

- Large-scale vaccination campaigns are often outbreak-driven, time-limited, and exclude pregnant populations due to safety concerns, and family planning messaging for this context should be developed and pilot-tested.
- Given the limited nature of programmatic data, we were unable to explore the role of predictors of family planning use such as partner information, frequency of sexual intercourse, or income which would be informative for designing family planning messaging for this context.

## Introduction

Ebola virus disease was first detected in Africa in the Democratic Republic of the Congo (DRC) in 1976. There have been regular outbreaks in recent years, the most recent occurring in August 2022 [1]. There is high risk associated with the disease, especially for pregnant women and their babies [2,3]. Ongoing outbreaks in DRC and other African countries have necessitated international collaboration, community education, and interventions to address outbreaks and enhance preparation [4,5].

As a country bordering DRC, Rwanda is particularly vulnerable. Prior to the COVID-19 pandemic, it was estimated that nearly 90,000 people crossed the Rwandan border with DRC each day [6]. Most of the cross-border traffic is informal, small-scale trade. Women make up 80% of agricultural produce traders, making it especially important to target this group in interventions [6].

In 2019, the Rwandan Food and Drug Administration (FDA) granted emergency use authorization for a heterologous, non-replicating, two-dose Ebola vaccine (Ad26.ZEBOV, MVA-BN-Filo) developed by Janssen Pharmaceuticals. Clinical studies demonstrated that the two-dose regimen induced a robust and durable immune response in healthy adult African volunteers for at least 360 days, contributing to Rwanda’s decision to grant emergency use authorization [7–9].

Between December 2019 to September 2021, the Rwandan UMURINZI (**U**nprecedented **M**ovement to drive a **U**nified **R**wandan **I**nitiative for **N**ational **Z**EBOVAC **I**mmunization) vaccine campaign vaccinated 216,114 Rwandan non-pregnant individuals aged ≥2 years living in close proximity to the DRC border. The campaign targeted 2 districts, Rusizi and Rubavu, due to high rates of cross-border travel. The campaign was implemented by the Center for Family Health Research (CFHR), a Rwandan non-governmental organization, and the Rwanda Biomedical Center (RBC), a Rwandan government organization. We previously described that the vaccination regimen was safe and well-tolerated in children, men, and non-pregnant women [10].

The WHO Strategic Advisory Group of Experts (SAGE) has stated that “…, the development of non-replicating Ebola vaccine candidates should proceed with priority, as they represent fewer safety concerns for use in pregnancy” [11]. No concerning safety signal was expected for persons vaccinated with Ad26.ZEBOV and MVA-BN-Filo during pregnancy or their neonates given: (1) the limited data on incidental vaccination during pregnancy in prior clinical trials did not raise safety concerns; (2) the vaccines are non-replicating and with no expected trans-placental transfer after intramuscular injection; (3) animal studies indicated limited biodistribution; (4) animal studies did not indicate risks to the fetus.

However, pregnant women were excluded from participation in UMURINZI due to limited vaccine safety data in pregnant women at the time. Since unplanned incident pregnancy after dose one could reduce the number of women who receive dose two, minimizing community protection against Ebola virus, especially for women, the UMURINZI campaign provided education and access to contraceptive methods. The Rwanda DHS 2019 to 2020 [12] reported that knowledge of modern contraception is almost universal in Rwanda among both women and men (99% to 100%), and most married women using contraception use a modern method (58%) [12]. Contraception is accessible at all non-Catholic-affiliated government-run health centers, while the roughly 60% of clinics in Rwanda affiliated with the Catholic church [13] do not provide contraception, they refer interested clients to nearby health posts which do provide counseling and methods [14].

There is no identifiable literature about offering family planning methods for short-term pregnancy prevention during multidose vaccination campaigns that exclude pregnant women. Here, we describe contraceptive use, pregnancy incidence, and serious adverse events (SAEs), as well as associations between pregnancy and SAE on the outcome of receipt of the second vaccine dose, among women in the campaign.

## Methods

### UMURINZI recruitment and eligibility

Vaccine recipients were recruited between December 2019 to September 2021 through local media advertisements; megaphone announcements in markets and villages; promotions at religious gatherings, workers associations, and monthly community service days; and community health workers (CHWs). Eligibility criteria included being a Rwanda resident, aged ≥2 years, healthy with no fever or history of severe allergies, not pregnant, and willing to return for a second vaccination dose. Recruited participants were referred to one of 14 vaccination sites within Rubavu and Rusizi districts which included 12 standing health centers and 2 tent facilities at the border crossing between Rubavu, Rwanda and Goma, DRC. Data from a small, mobile vaccination clinic at the Kigali airport are not included in these analyses. Two of the 14 vaccination sites were co-located with Catholic-affiliated health facilities; one provided family planning to vaccination campaign participants while the other referred interested women to the nearby health post. The campaign aimed to reach at least 200,000 Rwandan individuals based on need, feasibility, and resources.

### Vaccine product

This vaccination campaign used the Janssen Vaccines & Prevention B.V. 2-dose heterologous Ebola vaccine regimen (Ad26.ZEBOV vaccine followed by MVA-BN-Filo vaccine 8 weeks later).

### Procedures at the first vaccination visit (baseline)

During the first vaccination, eligibility, demographic, and fertility status data were recorded for women and girls. A urine pregnancy test was conducted, and current contraceptive use was recorded for those aged 15 to 50 years and menarchal. Eligible vaccinees underwent iris scanning linked with real-time cloud-based data to track the first and second dose to the same participant regardless of vaccination site [15]. Results were documented in logbooks and data were entered in SurveyCTO.

Before entering the vaccination room, all participants received a group information session on the Ebola vaccines to be used, the requirement that only non-pregnant persons were eligible, and that there would be an opportunity to discuss and receive family planning information and methods in order to prevent pregnancy. Providing this information to male as well as female participants was done so that men would understand why some partners were ineligible for vaccination (after a positive pregnancy test) and also to increase support for their partners if they chose to start a family planning method at the vaccination site.

In each vaccination facility, close linkages between our vaccination campaign and family planning services were instituted in the weeks after vaccination services were streamlined.

UMURINZI vaccination nurses provided one-on-one family planning counselling to fertile women who were not using any family planning method before vaccination during the 30-min observation waiting period after vaccination. Topics included: (1) recommendations to prevent pregnancy between doses to ensure receipt of both doses for maximum protection; (2) discussion of the benefits of family planning to those who reported wanting to space/limit childbearing; and (3) information on all reversible family planning methods (IUD, implants, injectables, and oral contraceptive pills) depending on the fertility intention of the participant. Based on the comparatively high typical-use failure rate of condoms and OCPs in the literature and our previous studies and programs promoting contraception in Rwanda [16–20], fertile women who were not using injectables, IUD, or an implant were offered additional contraceptive counseling. Condoms were provided to those who did not choose to use any other family planning method. Dual protection was offered if participants voluntarily disclosed that they were living with HIV. Family planning brochures briefly describing all family planning methods which are used routinely in government clinics were given to women after family planning counselling. For women attending the vaccination with their partners and who wanted to involve partners in family planning decisions, family planning counselling was offered jointly to the couple. Vaccinees at health centers were referred to co-located family planning services for receipt of same-day contraception before leaving the vaccination site. Vaccinees at the tent facility were provided with contraceptives by trained vaccination program nurses.

### Procedures at the second vaccination visit

Iris scanning and pregnancy tests were repeated at presentation for the second dose. In the case of a positive pregnancy test, women were referred for co-located antenatal care services (or for tent facility attendees, a nearby health center). A registry of women with an incident pregnancy detected at the second dose appointment was maintained, and they were contacted regularly by phone or in-person to assess pregnancy outcomes. Results from the routine first antenatal care visit obstetric ultrasound are recorded on each woman’s antenatal card and include estimated date of delivery. Women were advised to let the vaccine program know when the pregnancy ended so an appointment could be made for the second dose. In the event of a pregnancy loss, details of the obstetric outcome were recorded including spontaneous abortion (losses occurring before 20 weeks gestation), stillbirth (losses occurring > = 20 weeks gestation), or abnormalities on ultrasound leading to induced pregnancy termination. Where indicated, health center or hospital records were consulted for more detail. Unfortunately, pregnant women who were still pregnant at the time the UMURINZI program ended were not able to receive the second dose.

### Adverse events reporting

Vaccinees were advised of potential local and systemic reactogenicity signs and symptoms and were provided with a phone number on their vaccination card to reach a program physician in the event symptom management, a clinic visit, or hospitalization were warranted. These phone calls and visits to vaccination sites were recorded in a separate logbook and entered into SurveyCTO as unsolicited adverse events (UAE). Hospitalizations and other SAEs including pregnancy loss were followed up by UMURINZI physicians and reported to regulatory authorities including the Rwandan FDA and the sponsor, Janssen [10].

### Adverse events relatedness determination

SAE were defined based on International Conference on Harmonization and European Union Guidelines on Pharmacovigilance for Medicinal Products for Human Use [21], a SAE was defined as any untoward medical occurrence that: results in death or fetal loss; is life-threatening; results in persistent or significant disability/incapacity; requires in-patient hospitalization or prolonging of existing hospitalization; is a congenital anomaly/birth defect in the offspring of a vaccination campaign participant; and/or is an important medical event that may jeopardize the participant or may require intervention to prevent one of the other outcomes listed above.

SAE relatedness to vaccine was assessed by physicians at both Districts and this was reviewed and agreed upon with the team of physician investigators centrally at CFHR. Relatedness was assessed using the safety background information available on the investigator’s brochures (which listed no adverse drug reactions or events of special concern including among 79 pregnant women inadvertently vaccinated with the regimen in prior campaigns), as well as using clinical judgment including consideration of temporality between vaccine and the SAE. Due to emerging concerns about Thrombotic Thrombocytopenia Syndrome (TTS) related to the Ad26.COV2.S COVID-19 vaccine, in July of 2021, the Janssen team led a training of the program physicians to alert them to potential risks related to TTS. There was no independent safety monitoring board for the UMURINZI campaign.

### Data analyses

The vaccination campaign was not designed to test any prespecified hypotheses, and there was no prospective statistical analysis plan developed prior to the campaign other than for routine reporting. The analyses presented here are data-driven.

Data from Survey CTO were imported, cleaned, and analyzed in SAS v9.4 (Cary, NC). Information collected from the first vaccination logbook established a denominator of women eligible to receive family planning services (aged 15 to 50 years, fertile, and sexually active), and contraceptive use at the first dose appointment and uptake of a new method after receiving the first dose is described. In this population, factors associated with contraceptive use between dose one and the dose two appointment as well as predictors of pregnancy incidence detected at the dose two appointment were evaluated using chi-square tests and multivariable logistic regression. Age was modeled as a polynomial in response to peer reviewer comment and since the relationship between age and outcomes of interest followed a polynomial distribution with 2 degrees. Adjusted odds ratios (aOR) and 95% confidence intervals (CIs) are calculated adjusting for age, district of vaccination, frequency of DRC border crossing. Attending a health center versus the dedicated vaccine tent was not included in the multivariable logistic model as it was collinear with district. We also describe obstetric and non-obstetric SAE/UAE by incident pregnancy status and receipt of dose two for women aged ≥15 years.

### Ethics

After review of the concept proposal describing UMURINZI (S1 Text), the vaccination campaign was determined to not be research by the Emory IRB and Rwanda National Ethics Committee (RNEC). The Rwandan FDA also reviewed the concept proposal and an UMURINZI Factsheet for vaccine recipients (S2 Text). Verbal consent was obtained from UMURINZI vaccine recipients prior to vaccination after a trained nurse verbally provided the details of the Rwanda FDA-approved Factsheet to the recipient.

### Reporting

This study is reported as per the REporting of studies Conducted using Observational Routinely collected Data (RECORD) guideline (S1 RECORD Checklist).

## Results

### Contraceptive use at baseline and uptake after dose one

**Fig 1:**
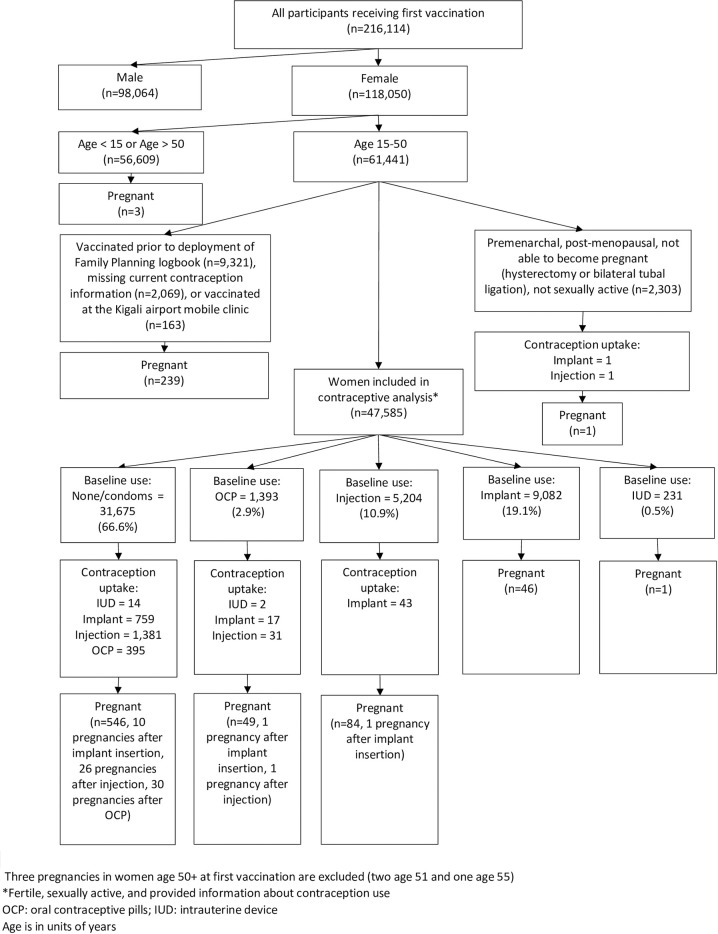
Flow diagram of contraception use, uptake after dose one, and pregnancy.

The first dose of Ebola vaccine was administered to 216,114 Rwandan individuals (Fig 1). Of these, 61,441 (28·4%) were women aged 15 to 50 years among whom 969 incident pregnancies occurred after women received dose one.

The analyses of contraception use, uptake, and pregnancy includes the 47,585 women and girls aged 15 to 50 years who were fertile, sexually active, and with contraceptive use data. Of these 47,585 fertile and sexually active women (S1 Table), the mean age was 28·0 years (standard deviation 9·9 years), 54·7% were from Rubavu and 45·3% were from Rusizi, and 71·9% had not crossed the DRC border in the last year. Of these women, 66·6% were not using contraception, 2·9% were using OCP, 10·9% injectables, 19·1% implant, and 0·5% copper IUD. After contraceptive counseling and offer of family planning, 2,549 (8·0%) of method non-users initiated a method including injectables (4·4%), implants (2·4%), and OCP (1·2%) (Fig 1). Among baseline OCP users, 3·6% switched to a more effective method including injectables (2·2%), implant (1·2%), and IUD (0·1%). A small number of injectable users chose hormonal implant (0·8%). Overall, the number of modern contraceptive users increased from 33·4% to 38·8%.

### Distribution and correlates of contraceptive use

S2 Table presents the distribution of contraception use between the dose one and dose two appointment by age (described using means and SD and modeled as a polynomial), district, frequency of border crossing into DRC, and vaccination facility type. Contraceptive users were significantly older than those using no contraceptives or condoms only: mean age for contraceptive users was 32·0 years (SD = 7·4) while the mean age of non-users was 25·5 years (SD = 10·4). Among contraceptive users, IUD users had the oldest mean age of 34·8 years (SD = 7·1), followed by OCP users (mean = 33·4 years, SD = 7·5), injectable users (mean = 32·7 years, SD = 7·4), and implant users (mean = 31·1 years, SD = 7·2). Contraceptive use was higher in Rubavu (42·7%) than Rusizi (34·0%). Implant use accounted for most of the difference between Rusizi (16·3%) and Rubavu (24·5%). Women who crossed the border into DRC in the last year were more likely versus those who did not cross the border to be using implants (25·5 to 28·7% versus 18·2%) and injectables (18·6% to 19·7% versus 11·7%).

Statistically significant (*p* < 0·05) correlates of modern contraceptive use in multivariate analysis included age (the adjusted odds ratio for the linear term of age modeled as a polynomial was aOR = 2·4; 95% CI = 2·4, 2·5; *p* < 0·0001 indicating that contraceptive use increased with age; the quadratic term of age squared indicated a slight decrease in the odds of the contraceptive use as age increased beyond a certain point; see S2 Table footnote); residing in Rubavu (aOR = 2·0; 95%CI = 1·9, 2·1; *p* < 0·0001) versus Rusizi; crossing the border into DRC at least once a week (aOR = 1·2, 95%CI = 1·1, 1·3; *p* < 0·0001) or less than once a week (aOR = 1·1; 95%CI = 1·0, 1·2; *p* = 0·0087) versus not. Each covariate is adjusted for every other covariate in the model.

### Distribution and correlates of incident pregnancy

Of the 726 pregnancies detected at the dose two appointment in the 47,585 women shown in Fig 1, 66·1% occurred in women who were not using contraception at baseline and did not adopt a method after family planning counseling; 10·6% occurred in OCP users, 15·2% in injectable users, 8·0% in implant users, and 0·1% in IUD users.

Pregnancy risk was highest in OCP users (4·4%), intermediate in non-users (1·6%), and injectable users (1·7%), and lowest in implant (0·6%) and IUD (0·4%) users (S3 Table). In multivariable analysis, relative to non-contraceptive users, OCP users were more likely to become pregnant (aOR = 1·4; 95%CI = 1·1, 1·9; *p* = 0·0042) while injectable and implant users were less likely (aOR = 0·6; 95%CI = 0·5, 0·7; *p* < 0·0001 and aOR = 0·2; 95%CI = 0·1, 0·3; *p* < 0·0001, respectively). Each covariate is adjusted for every other covariate in the model.

Pregnancy risk was higher in women initiating an implant after dose one counseling compared to those already using an implant at baseline (1·5% versus 0·5%, *p* = 0·0024); similarly, pregnancy risk was higher in women initiating OCPs after dose one counseling compared to those already using OCPs at baseline (7·6% versus 3·5%, *p* = 0·0005) (S4 Table).

### Adverse events and receipt of second dose by pregnancy status

**Fig 2:**
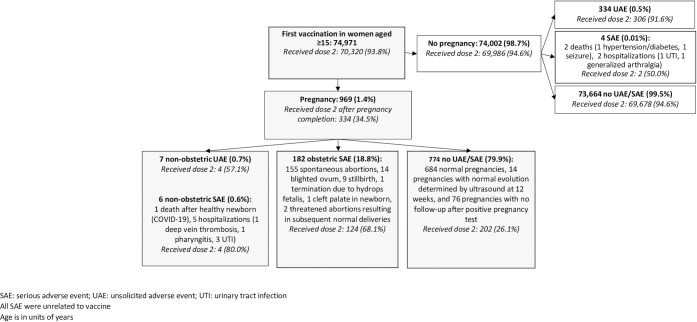
Serious adverse events, unsolicited adverse events, and receipt of dose two by pregnancy status.

Of the 74,971 women aged ≥15 years receiving dose one, 969 (1·4%) became pregnant between the first and second dose appointments which occurred a median 58 days apart (range 25 to 616 days) (Fig 2). Among women without a pregnancy, 94·6% received the second dose; in contrast, only 34·5% of women with a pregnancy received dose two after pregnancy completion.

Of women with incident pregnancy after dose one, 182 (18·8%) had an obstetric SAE including 155 spontaneous abortions, 14 blighted ova, 9 stillbirths, 1 termination due to hydrops fetalis detected on routine ultrasound, 1 cleft palate in the newborn, and 2 threatened abortions resulting in subsequent normal deliveries. The overall fetal loss proportion was 18·5%. Receipt of the second dose after pregnancy completion was documented in 68·1% of women experiencing an obstetric SAE. Only 6 pregnant women had a non-obstetric SAE (0·6%), including 1 postpartum death from COVID-19 and 5 hospitalizations (1 deep vein thrombosis, 2 pharyngitis, and 3 urinary tract infections). Four of the 5 hospitalized women received their second dose. The remaining 774 pregnant women included 684 reported deliveries, 14 normal ultrasound findings in the first trimester and no further follow-up, and 76 with no follow-up after the pregnancy test prior to dose two. Of these 774 women, only 26·1% received their second dose.

Among women with no pregnancy, 334 (0·5%) had a UAE and 4 (0·01%) had an SAE. Two SAE were fatalities due to longstanding hypertension and diabetes in one case and seizures in the other. The other 2 SAE were hospitalizations for urinary tract infection and generalized arthralgia, and both women received the second dose after recovery. Receipt of dose two was recorded for 91·6% of women reporting UAE and 94·6% with no adverse events.

No SAE were determined to be related to the vaccine by the program physicians. The median number of days between vaccination and obstetric SAE was 95 days (range 6 to 468 days). The SAE occurring 6 days after vaccination was a threatened abortion resulting in a subsequent normal delivery. After excluding this participant, the median number of days between vaccination and obstetric SAE was 95 days (range 26 to 468 days).

## Discussion

The large-scale UMURINZI Ebola vaccination campaign presented a unique opportunity to analyze family planning use, pregnancy incidence and outcomes, and completion of the two-dose vaccine regimen in an area with high rates of pregnancy. In summary, 66·6% of fertile and sexually active women were not using contraception at the time of their first vaccine dose, and only 8·0% of these initiated a new method after the provision of family planning counseling and contraceptives. Spontaneous abortion occurred in 16·0% of pregnancies. No SAE were related to vaccine. Finally, in the 2 months between the first and second dose, 969 women became pregnant of whom only 34·5% received their second dose, thus potentially reducing protection against Ebola virus disease at a particularly vulnerable time. Meanwhile, 94·6% of women who did not become pregnant received their second dose.

Risk of spontaneous abortion in women who became pregnant after receiving their first vaccine dose in UMURINZI was 16·0%. In population-level studies, spontaneous abortion risk has been estimated to range between 8% to 33% in Africa [22–25] and 7% to 21% in western countries [26–32]. The most recent prior estimate of spontaneous abortion in Rwanda, we identified was derived from data collected roughly 15 years ago which estimated that 16% of pregnancies ended in miscarriage by back calculating from the estimated number of abortions, births, and the expected patterns of miscarriages from the medical literature [22].

Most incident pregnancies occurred in OCP users. OCPs are one of the least effective modern methods with typical use [33,34] due to missing daily doses, barriers to refilling pills, and side effects. Pregnancies among injectable users might have been due to missing a quarterly injection, which may have been exacerbated by health center closures and quarantine regulations that accompanied the COVID-19 epidemic. In a few cases, very early pregnancies resulting in a false negative pregnancy test at the time of the dose one visit may have occurred. For example, within the first month after dose one, one threatened abortion (6 days after dose one) and one spontaneous abortion (26 days after dose one) occurred, and these events may have occurred after a false negative pregnancy test. Within the second month after dose one (32 to 60 days after dose one), 11 spontaneous abortions and 3 blighted ova occurred. As expected, long-acting reversible contraceptives (LARCs) were associated with low pregnancy incidence.

Older women were more likely to use contraceptives than younger women, and they were also more likely to have an incident pregnancy. We postulate that older women are likely to be in cohabiting partnerships and having more frequent sex. In the southern Rusizi district, which has a larger rural community, contraceptive use was lower than in Rubavu (34·0% versus 42·7%) and pregnancy risk was higher (2·1% versus 1·1%); these trends are expected based on previous data of urban-rural differences in contraception use and pregnancy in Rwanda [35].

Uptake of contraception in UMURINZI after counseling was low after the provision of family planning counseling and contraceptives. Prior family planning research and implementation studies in Rwanda, including among rural and Catholic clients [16–20,36–40], and Zambia [18,33] conducted by CFHR indicate that when counselling messages have been adapted to address common questions and concerns, community-based education is provided, and when male partners are involved in discussions, contraceptive method uptake increases. However, the context of a vaccination campaign, which necessitated only short-term use of contraception, is a different context compared to this prior work which primarily delivered contraceptive education to meet the fertility goals of women and couples seeking care at public heath family planning clinics, antenatal care, and infant vaccination or in the context of research studies [16–20,33,36–40]. To the best of our knowledge, counseling to prevent pregnancy in a very short interval to ensure complete vaccination is a new concept.

Studies to identify the specific needs of a population of women seeking vaccination could be highly informative for tailoring messages for future vaccination campaigns. For example, future work could consider creating counseling flipcharts for this context which discuss (1) pregnancy-related risks associated with contracting Ebola during pregnancy; (2) considering options for short-term contraception, for example, via receipt of one injection of hormonal contraceptive specifically to cover the time frame between the first and second dose; and (3) considering LARC methods for those who want to prevent pregnancy longer-term. New method adopters should be advised to use another contraceptive method for the first 2 weeks after, as evidenced by the higher pregnancy risk among new OCP and implant adopters compared with existing users in UMURINZI.

Additionally, future work could consider pre-campaign messaging and sensitization delivered via CHW or mass media that describes the availability of and reasons for family planning offered in the context of the campaign. Couples could also be encouraged to come together for vaccination to enhance uptake as previous research has demonstrated the importance of male partner involvement in family planning decision-making in Rwanda [18,19,41,42].

Finally, UMURINZI was a time-limited, urgent campaign and many of the pregnancies did not end before the campaign ended. Future two-dose campaigns should be designed to ensure that women who become pregnant are able to get their second dose after pregnancy completion.

These findings and recommendations have implications for future mass vaccine and drug administrations which exclude pregnant women due to a lack of safety data [43]. For example, public health prevention and treatment programs for cholera, schistosomiasis, onchocerciasis, and lymphatic filariasis often exclude pregnant women due to limited safety data or drug safety concerns [44]. The current fertility rate in Rwanda is 3·9, 28% of women of reproductive age are pregnant within any given year, and half of pregnancies in Rwanda are unintended; thus many women spend much of their reproductive life either pregnant or breastfeeding, limiting the opportunity for many women to participate in or receive the full benefit of health campaigns that exclude pregnant women due to a lack of safety data [35].

More evidence regarding Ebola vaccine safety in pregnant populations is needed to inform guidelines. Under a clinical trial protocol, >20,000 people from DRC were vaccinated during the 2019 to 2020 Ebola outbreak in the DRC including >800 pregnant women who were not excluded from the program due to the greater benefit-risk ratio during an active outbreak. However, no rigorous study of safety data among pregnant women could be conducted due to the challenging conditions on the ground in DRC, including unstable security and lack of developed healthcare system infrastructure. CFHR recently completed a randomized trial of safety and immunogenicity of the two-dose Ad26.ZEBOV, MVA-BN-Filo used in UMURINZI among pregnant women in the same high-risk border regions [45], and results are forthcoming.

Some limitations warrant consideration. Many clinics and vaccination sites did not have adequate staff training, infrastructure, or supplies to consistently offer IUD. OCP use was based on self-report. Though pregnancy data were primarily self-reported, the requested information is recorded on women’s antenatal care cards which they retain. Given the limited nature of programmatic data, we were unable to explore the role of factors such as partner information, sexual intercourse frequency, or income. Finally, though we are missing contraceptive information for a relatively small proportion of women due mainly to the delay in instituting the family planning logbook after vaccination sites were launched, it is unlikely that those excluded are systematically different.

Multidose vaccination campaigns that exclude pregnant women should provide family planning counseling and method provision to improve gender equity and community protection against vaccine-preventable diseases. Family planning messaging for this context should be developed and pilot tested, including counseling about pregnancy risk shortly after method initiation.

## Supporting information

S1 Table Distribution of baseline characteristics of fertile, sexually active, women who sought vaccination during UMURINZI (*n* = 47,585). (DOCX)

S2 Table Distribution and correlates of contraceptive method use between dose one and dose two appointment (*n* = 47,585). (DOCX)

S3 Table Distribution and correlates of incident pregnancy detected at dose two appointment (*n* = 47,585). (DOCX)

S4 Table Pregnancy risk in baseline method users versus new initiators. (DOCX)

S1 Text UMURINZI concept proposal. (PDF)

S2 Text UMURINZI Factsheet. (PDF)

S1 RECORD Checklist The RECORD statement checklist. (PDF)

## Abbreviations

aOR: adjusted odds ratio
CHW: community health worker
CI: confidence interval
DRC: Democratic Republic of the Congo
FDA: Food and Drug Administration
LARC: long-acting reversible contraceptive
OCP: oral contraceptive pill
RBC: Rwanda Biomedical Center
SAE: serious adverse events
SAGE: Strategic Advisory Group of Experts
TTS: Thrombotic Thrombocytopenia Syndrome
UAE: unsolicited adverse event
