# Correction: Contraception use and pregnancy in women receiving a 2-dose Ebola vaccine in Rwanda: A retrospective analysis of UMURINZI vaccination campaign data

**DOI:** 10.1371/journal.pmed.1004605

**Published:** 2025-04-30

**Authors:** The PLOS Medicine Editors

**Notice of Republication**

This article [1] was republished on April 11, 2025, to address an issue identified post-publication. Please download this article again to view the correct version.
