# Supplementary material for: Contraception use and pregnancy in women receiving a 2-dose Ebola vaccine in Rwanda: A retrospective analysis of UMURINZI vaccination campaign data
Source: PLoS Med. 2025 Feb 11;22(2):e1004508. doi: 10.1371/journal.pmed.1004508 (PMC11813098; doi:10.1371/journal.pmed.1004508)
Supplement: S1 Table — (DOCX) [file pmed.1004508.s001.docx]

| **S1 Table.** Distribution of baseline characteristics of fertile, sexually active, women who sought vaccination during UMURINZI (n=47,585) | | | | | |
| --- | --- | --- | --- | --- | --- |
|  | Total (n=47585) | |  |  |  |
|  | n/mean | Col %/SD |  |  |  |
| **Age** | 28.0 | 9.9 |  |  |  |
| **District** |  |  |  |  |  |
| Rubavu | 26051 | 54.7% |  |  |  |
| Rusizi | 21534 | 45.3% |  |  |  |
| **Crossed DRC border in the last year** |  |  |  |  |  |
| Yes at least 1/week | 8620 | 18.1% |  |  |  |
| Yes <1/week | 4721 | 9.9% |  |  |  |
| No | 34158 | 71.9% |  |  |  |
| **Vaccination facility** |  |  |  |  |  |
| Health center | 46449 | 97.6% |  |  |  |
| Dedicated vaccine facility (tent) | 1136 | 2.4% |  |  |  |
| **Contraceptive use at baseline** |  |  |  |  |  |
| None | 31675 | 66.6% |  |  |  |
| OCP | 1393 | 2.9% |  |  |  |
| Injectable | 5204 | 10.9% |  |  |  |
| Implant | 9082 | 19.1% |  |  |  |
| IUD | 231 | 0.5% |  |  |  |
| OCP: oral contraceptive pills; IUD: intrauterine device; DRC: Democratic Republic of the Congo; SD: standard deviation  Age is in units of years | | | | |  |
