# Supplementary material for: Contraception use and pregnancy in women receiving a 2-dose Ebola vaccine in Rwanda: A retrospective analysis of UMURINZI vaccination campaign data
Source: PLoS Med. 2025 Feb 11;22(2):e1004508. doi: 10.1371/journal.pmed.1004508 (PMC11813098; doi:10.1371/journal.pmed.1004508)
Supplement: S2 Table — (DOCX) [file pmed.1004508.s002.docx]

| **S2 Table.** Distribution and correlates of contraceptive method use between dose one and dose two appointment (n=47,585) | | | | | | | | | | | | | | | | | |  |  |  |  |  |  |  |  |
| --- | --- | --- | --- | --- | --- | --- | --- | --- | --- | --- | --- | --- | --- | --- | --- | --- | --- | --- | --- | --- | --- | --- | --- | --- | --- |
|  | **Distribution of contraceptive use** | | | | | | | | | | | | | | | | **Unadjusted logistic regression models** | | | | **Adjusted logistic regression model** | | | |  |
|  | None/Condoms (n=29126) | | OCP  (n=1738) | | Injectable (n=6573) | | Implant  (n=9901) | | IUD  (n=247) | |  | None/Condoms (n=29126) | | Any contraceptive  (n=18459) | |  | Any contraceptive use versus none/condoms | | | | Any contraceptive use versus none/condoms | | | |  |
|  | n/ mean | Row %/SD | n/ mean | Row %/SD | n/ mean | Row %/SD | n/ mean | Row %/SD | n/ mean | Row %/SD | p-value | n/ mean | Row %/SD | n/ mean | Row %/SD | p-value | cOR | 95% CI | | p-value | aOR | 95% CI | | p-value |  |
| **Age (linear term of polynomial)*** | 25.5 | 10.4 | 33.4 | 7.5 | 32.7 | 7.4 | 31.1 | 7.2 | 34.8 | 7.1 | <0.0001 | 25.5 | 10.4 | 32.0 | 7.4 | <0.0001 | 2.4 | 2.4 | 2.4 | <0.0001 | 2.4 | 2.4 | 2.5 | <0.0001 |  |
| **District** |  |  |  |  |  |  |  |  |  |  |  |  |  |  |  |  |  |  |  |  |  |  |  |  |  |
| Rubavu | 14921 | 57.3% | 673 | 2.6% | 3948 | 15.2% | 6387 | 24.5% | 122 | 0.5% | <0.0001 | 14921 | 57.3% | 11130 | 42.7% | <0.0001 | 1.5 | 1.4 | 1.5 | <0.0001 | 2.0 | 1.9 | 2.1 | <0.0001 |  |
| Rusizi | 14205 | 66.0% | 1065 | 4.9% | 2625 | 12.2% | 3514 | 16.3% | 125 | 0.6% |  | 14205 | 66.0% | 7329 | 34.0% |  | ref |  |  |  | ref |  |  |  |  |
| **Crossed DRC border in the last year** |  |  |  |  |  |  |  |  |  |  |  |  |  |  |  |  |  |  |  |  |  |  |  |  |  |
| Yes at least 1/week | 4043 | 46.9% | 442 | 5.1% | 1600 | 18.6% | 2478 | 28.7% | 57 | 0.7% | <0.0001 | 4043 | 47.2% | 4520 | 52.8% | <0.0001 | 2.3 | 2.2 | 2.4 | <0.0001 | 1.2 | 1.1 | 1.3 | <0.0001 |  |
| Yes <1/week | 2311 | 49.0% | 239 | 5.1% | 931 | 19.7% | 1202 | 25.5% | 38 | 0.8% |  | 2311 | 49.3% | 2372 | 50.7% |  | 2.1 | 2.0 | 2.2 | <0.0001 | 1.1 | 1.0 | 1.2 | 0.0087 |  |
| No | 22769 | 66.7% | 1039 | 3.0% | 3995 | 11.7% | 6204 | 18.2% | 151 | 0.4% |  | 22769 | 66.7% | 11389 | 33.3% |  | ref |  |  |  | ref |  |  |  |  |
| **Vaccination facility** |  |  |  |  |  |  |  |  |  |  |  |  |  |  |  |  |  |  |  |  |  |  |  |  |  |
| Health center | 28441 | 61.2% | 1654 | 3.6% | 6436 | 13.9% | 9684 | 20.8% | 234 | 0.5% | <0.0001 | 28441 | 61.2% | 18008 | 38.8% | 0.5245 | 1.0 | 0.9 | 1.1 | 0.5228 |  |  |  |  |  |
| Dedicated vaccine facility (tent) | 685 | 60.3% | 84 | 7.4% | 137 | 12.1% | 217 | 19.1% | 13 | 1.1% |  | 685 | 60.3% | 451 | 39.7% |  | ref |  |  |  |  |  |  |  |  |
| OCP: oral contraceptive pills; IUD: intrauterine device; DRC: Democratic Republic of the Congo; SD: standard deviation; cOR: crude odds ratio; aOR: adjusted odds ratio; CI: confidence interval | | | | | | | | | | | | | | | | | | | | |  |  |  |  |  |
| p-values (from Chi-square tests for categorical variables and t-test for continuous variables) are two-sided | | | | | | | | | | | | |  |  |  |  |  |  |  |  |  |  |  |  |  |
| *The aOR for the quadratic term (age squared) of age modeled as a polynomial is aOR = 0.98699; 95%CI = 0.98668-0.98729, p <0.001 | | | | | | | | | | | | | | |  |  |  |  |  |  |  |  |  |  |  |
| Age is in units of years |  |  |  |  |  |  |  |  |  |  |  |  |  |  |  |  |  |  |  |  |  |  |  |  |  |
| Adjusted model includes age, district, crossed DRC border | | | | | | | |  |  |  |  |  |  |  |  |  |  |  |  |  |  |  |  |  |  |
