# Supplementary material for: Contraception use and pregnancy in women receiving a 2-dose Ebola vaccine in Rwanda: A retrospective analysis of UMURINZI vaccination campaign data
Source: PLoS Med. 2025 Feb 11;22(2):e1004508. doi: 10.1371/journal.pmed.1004508 (PMC11813098; doi:10.1371/journal.pmed.1004508)
Supplement: S3 Table — (DOCX) [file pmed.1004508.s003.docx]

| **S3 Table.** Distribution and correlates of incident pregnancy detected at dose two appointment (n=47,585) | | | | | | |  |  |  |  |  |  |  |
| --- | --- | --- | --- | --- | --- | --- | --- | --- | --- | --- | --- | --- | --- |
|  | **Distribution of incident pregnancy detected at dose two appointment** | | | | | **Unadjusted logistic regression models** | | | | **Adjusted logistic regression model** | | | |
|  | Incident pregnancy | | | |  | Incident pregnancy yes versus no | | | | Incident pregnancy yes versus no | | | |
|  | Yes  (n=726) | | No  (n=46859) | | p-value | cOR | 95% CI | | p-value | aOR | 95% CI | | p-value |
| **Contraceptive use after baseline** | n/mean | Row %/SD | n/mean | Row %/SD |  |  |  |  |  |  |  |  |  |
| None (n=29,126) | 480 | 1.6% | 28646 | 98.4% | <0.0001 | ref |  |  |  | ref |  |  |  |
| OCP (n=1,738) | 77 | 4.4% | 1661 | 95.6% |  | 2.8 | 2.2 | 3.5 | <0.0001 | 1.4 | 1.1 | 1.9 | 0.0042 |
| Injectable (n=6,573) | 110 | 1.7% | 6463 | 98.3% |  | 1.0 | 0.8 | 1.3 | 0.8835 | 0.6 | 0.5 | 0.7 | <0.0001 |
| Implant/IUD* (n=10,148) | 59 | 0.6% | 10089 | 99.4% |  | 0.4 | 0.3 | 0.5 | <0.0001 | 0.2 | 0.1 | 0.3 | <0.0001 |
| **Age (linear term of polynomial)**** | 30.0 | 7.7 | 28.0 | 9.9 | <0.0001 | 1.6 | 1.5 | 1.7 | <0.0001 | 1.8 | 1.7 | 1.8 | <0.0001 |
| **District** |  |  |  |  |  |  |  |  |  |  |  |  |  |
| Rubavu (n=26,051) | 278 | 1.1% | 25773 | 98.9% | <0.0001 | ref |  |  |  | ref |  |  |  |
| Rusizi (n=21,534) | 448 | 2.1% | 21086 | 97.9% |  | 2.0 | 1.7 | 2.3 | <0.0001 | 1.6 | 1.3 | 1.8 | <0.0001 |
| **Crossed DRC border in the last year** |  |  |  |  |  |  |  |  |  |  |  |  |  |
| Yes at least 1/week (n=8,620) | 168 | 1.9% | 8452 | 98.1% | 0.0009 | 1.4 | 1.2 | 1.7 | 0.0002 | 1.2 | 1.0 | 1.4 | 0.0960 |
| Yes <1/week (n=4,721) | 78 | 1.7% | 4643 | 98.3% |  | 1.2 | 0.9 | 1.5 | 0.1816 | 1.1 | 0.8 | 1.4 | 0.5376 |
| No (n=34,158) | 480 | 1.4% | 33678 | 98.6% |  | ref |  |  |  | ref |  |  |  |
| **Vaccination facility** |  |  |  |  |  |  |  |  |  |  |  |  |  |
| Health center (n=46,449) | 709 | 1.5% | 45740 | 98.5% | 0.8747 | 1.0 | 0.6 | 1.5 | 0.8676 |  |  |  |  |
| Dedicated vaccine facility (tent) (n=1,136) | 18 | 1.6% | 1118 | 98.4% |  | ref |  |  |  |  |  |  |  |
| OCP: oral contraceptive pills; IUD: intrauterine device; DRC: Democratic Republic of the Congo; SD: standard deviation; cOR: crude odds ratio; aOR: adjusted odds ratio; CI: confidence interval | | | | | | | | | | | | |  |
| p-values (from Chi-square tests for categorical variables and t-test for continuous variables) are two-sided | | | | | | |  |  |  |  |  |  |  |
| *n=1 pregnancy among IUD users |  |  |  |  |  |  |  |  |  |  |  |  |  |
| **The aOR for the quadratic term (age squared) of age modeled as a polynomial is aOR = 0.99103; 95%CI = 0.98985-0.99221, p <0.001 | | | | | | | | |  |  |  |  |  |
| Age is in units of years |  |  |  |  |  |  |  |  |  |  |  |  |  |
| Adjusted model includes contraceptive use after baseline, age, district, crossed DRC border | | | | | |  |  |  |  |  |  |  |  |
