# Supplementary material for: Contraception use and pregnancy in women receiving a 2-dose Ebola vaccine in Rwanda: A retrospective analysis of UMURINZI vaccination campaign data
Source: PLoS Med. 2025 Feb 11;22(2):e1004508. doi: 10.1371/journal.pmed.1004508 (PMC11813098; doi:10.1371/journal.pmed.1004508)
Supplement: S4 Table — (DOCX) [file pmed.1004508.s004.docx]

**S4 Table.** Pregnancy risk in baseline method users versus new initiators

|  | **Baseline method use among those who did not initiate a more effective method** | | | | **Method initiators** | | | |  |
| --- | --- | --- | --- | --- | --- | --- | --- | --- | --- |
|  | Incident pregnancy | | No pregnancy | | Incident pregnancy | | No pregnancy | |  |
|  | n | Row % | n | Row % | n | Row % | n | Row % | p-value |
| None/condoms | 480 | 1.6% | 28646 | 98.4% | -- | -- | -- | -- | -- |
| OCP | 47 | 3.5% | 1296 | 96.5% | 30 | 7.6% | 365 | 92.4% | 0.0005 |
| Injectable | 83 | 1.6% | 5078 | 98.4% | 27 | 1.9% | 1385 | 98.1% | 0.4301 |
| Implant | 46 | 0.5% | 9036 | 99.5% | 12 | 1.5% | 807 | 98.5% | 0.0024 |
| IUD | 1 | 0.4% | 230 | 99.6% | 0 | 0.0% | 16 | 100.0% | 0.9352 |

OCP: oral contraceptive pills; IUD: intrauterine device

p-values (from Chi-square tests) are two-sided
