# Supplementary material for: Contraception use and pregnancy in women receiving a 2-dose Ebola vaccine in Rwanda: A retrospective analysis of UMURINZI vaccination campaign data
Source: PLoS Med. 2025 Feb 11;22(2):e1004508. doi: 10.1371/journal.pmed.1004508 (PMC11813098; doi:10.1371/journal.pmed.1004508)
Supplement: S1 Text — (PDF) [file pmed.1004508.s005.pdf]

**Program Title:**	**UMURINZI Ebola Vaccination Campaign (Unprecedented Movement to drive a Unified Rwandan Initiative for National ZEBOVAC Immunization)**
**Responsible Party:**	Ministry of Health<br>Republic of Rwanda<br>PO Box 84 Kigali<br>Rwanda
**Collaborating Organizations:**	Projet San Francisco/Center for Family Health Research<br>KK 19 Av 57<br>PO Box 780 Kigali<br>Rwanda<br><br>Rwanda Biomedical Center<br>PO Box<br>Rwanda<br><br>Emory University School of Medicine<br>101 Woodruff Circle, Suite 7313<br>Atlanta GA 30322<br>USA<br><br>L.E.A.F. Rwanda<br>RSSB Building Tower 2<br>Kigali<br>Rwanda<br><br>Rinda Ubuzima<br><br>Janssen Vaccines and Prevention B. V. (Janssen)<br>Turnhoutseweg 30, 2340 Beerse<br>Belgium
**Date of Current Version of the Concept Proposal**	1 October 2019
**Version:**	3.0

SYNOPSIS

The Statement on the meeting of the International Health Regulations (2005) Emergency Committee for Ebola virus disease in the Democratic Republic of the Congo on 17 July 2019 has recommended that at risk-countries “put in place approvals for investigational medicines and vaccines as an immediate priority for preparedness”. Following this recommendation, and after reviewing the application license, Rwanda FDA has granted conditional approval of the Ad26.ZEBOV and MVA-BN-Filo Ebola vaccine under exceptional emergency circumstances as part of an initiative to protect Rwandan citizens against Ebola.

With support from the Rwanda Ministry of Health, Projet San Francisco/Center for Family Health Research, Rwanda Biomedical Center and Rinda Ubuzima are implementing, in collaboration with Emory University, L.E.A.F. Rwanda, and Janssen Vaccines and Prevention B. V. (Janssen), the UMURINZI Ebola Vaccination Campaign (**U**nprecedented **M**ovement to drive a **U**nified **R**wandan **I**nitiative for **N**ational **Z**EBOVAC **I**mmunization). This is a large-scale population-based program to deliver the Ebola vaccine regimen VAC52150 (Ad26.ZEBOV and MVA-BN®-Filo) to adults, adolescents and children aged 2 years or over living in the vicinity of an Ebola outbreak.

The objective of the vaccination campaign is to provide the Ad26.ZEBOV, MVA-BN-Filo Ebola vaccine regimen to at least 193,000 people living in the vicinity of an Ebola outbreak.

The regimen consists of vaccination with Ad26.ZEBOV ($5x10^{10}$ viral particles (vp)) followed by a vaccination with MVA-BN-Filo ($1x10^{8}$ infectious units (Inf U)) approximately 8 weeks later.

Ad26.ZEBOV is a monovalent vaccine expressing the full-length glycoprotein (GP) from Ebola virus (EBOV) Mayinga. The vaccine is produced in the human cell line PER.C6®.

MVA-mBN226B, further referred to as Modified Vaccinia Ankara (MVA)-BN®-Filo, is a multivalent vaccine expressing the EBOV GP, the Sudan virus (SUDV) GP, the Marburg virus (MARV) Musoke GP, and the Taï Forest virus (TAFV, formerly known as Côte d’Ivoire ebolavirus) nucleoprotein (NP). The EBOV GP expressed by MVA-BN-Filo has 100% homology with the one expressed by Ad26.ZEBOV.

ABBREVATIONS

Ad26.ZEBOV	Adenovirus serotype 26 expressing the Ebola virus Mayinga glycoprotein
BN	Bavarian Nordic GmbH
CAC	Community Advisory Committee
DRC	The Democratic Republic of the Congo
EBOV	Ebola virus
ELISA	Enzyme-Linked ImmunoSorbent Assay
EMA	European Medicines Agency
ETC	Ebola Treatment Centre
EUA	Emergency Use Authorization
EVD	Ebola virus disease
FANG	Filovirus Animal Nonclinical Group
FDA	US Food and Drug Administration
FLW	FrontLine Worker
GCP	Good Clinical Practice
GP	GlycoProtein
HIV	Human Immunodeficiency Virus
ICH	International Council for Harmonization of Technical Requirements for Pharmaceuticals for Human Use
ICMJE	International Committee of Medical Journal Editors
IM	Intra Muscular
IND	Investigational New Drug
Inf U	Infectious Units
INRB	Institut National de Recherche Biomédicale
MHRA	Medicines and Healthcare Products Regulatory Agency
MOH	Ministry of Health, The Democratic Republic of the Congo
MVA	Modified Vaccinia Ankara
MVA-BN-Filo	Modified Vaccinia Ankara Bavarian Nordic vector expressing multiple filovirus proteins
NHP	Non-Human Primate(s)
PCR	Polymerase chain reaction
SAE	Serious Adverse Event
SAGE	WHO Strategic Advisory Group of Experts
SUSAR	Suspected Unexpected Serious Adverse Reaction
Vp	Viral Particles
WHO	World Health Organization

BACKGROUND

The statement on the meeting of the International Health Regulations (2005) Emergency Committee for Ebola virus disease (EVD) in the Democratic Republic of the Congo (DRC) on 17 July 2019 has recommended that at risk-countries “put in place approvals for investigational medicines and vaccines as an immediate priority for preparedness”. Following this recommendation, and after reviewing the application license submitted by Janssen Vaccines and Prevention B. V. (Janssen), on 27 September 2019, Rwanda FDA granted conditional approval of the Ad26.ZEBOV and MVA-BN-Filo Ebola vaccine under exceptional emergency circumstances as part of an initiative to protect Rwandan citizens against Ebola.

With support from the Rwanda Ministry of Health (MOH), Projet San Francisco/Center for Family Health Research (PSF/CFHR), Rwanda Biomedical Center (RBC) and Rinda Ubuzima are implementing, in collaboration with Emory University, L.E.A.F. Rwanda, and Janssen), the UMURINZI Ebola Vaccine Initiative (**U**nprecedented **M**ovement to drive a **U**nified **R**wandan **I**nitiative for **N**ational **Z**EBOVAC **I**mmunization). This is a large-scale population-based program to deliver the Ebola vaccine regimen VAC52150 (Ad26.ZEBOV and MVA-BN®-Filo) to adults, adolescents and children aged 2 years or over living in the vicinity of an Ebola outbreak.

Ebola Virus Disease

Ebola Virus Disease (EVD) is an acute systemic febrile syndrome caused by Ebola viruses. Ebola viruses belong to the Filoviridae family, characterized by their long and filamentous shape. EVD has a case fatality ranging from 30% to 90% and an incubation period of 2 to 21 days.[1] The disease-to-infection ratio is generally described as being 1:1 but some EVD infections may also be asymptomatic.[2] The highest case fatality on recent record, at 70%, was observed with Ebola virus (EBOV; formerly Zaire ebolavirus), the virus strain that caused the 2013-16 outbreak in West Africa.[3]

The pathogenesis of EVD is characterized by an intense inflammatory process, impaired hemostasis, and capillary leak, with mortality resulting from septic shock and multi-organ system failure.[1] Initial signs and symptoms are nonspecific (fever, headache, myalgia, fatigue) and may mimic more common conditions such as malaria. EVD progresses with gastrointestinal symptoms, internal and external bleeding, and in some cases, rash and neurologic involvement. Bats are suspected to be the reservoir for EBOV, although this remains to be confirmed.

Human infection is believed to result from contact with excrement or saliva from bats or other infected animals, followed by human-to-human transmission.[4, 5] In more than half of outbreaks, the animal origin of the virus has not been identified. EVD spreads by direct contact with body fluids of symptomatic patients. The virus can persist in the semen for up to a year or more after acute disease and be spread sexually.[6] The majority of infectious contacts are with close family members living in the same household. Contacts at funerals are also important for EVD transmission, where exposure may be through kissing, washing and touching the deceased. Moreover, frontline workers (FLW), including healthcare providers (HCP), members of burial teams and community outreach teams are at increased risk of contracting EVD. During the early phase of the 2013-16 outbreak in West Africa, a study in Guinea found that up to 38% of the EVD patients were HCP, with nosocomial transmission likely in 12 out of 14 cases.[7]

Ebola Outbreaks in DRC

Since Ebola virus was discovered in the Democratic Republic of the Congo (DRC; formerly Zaire) in 1976, the country has recorded ten Ebola outbreaks in total, more than any other country. The ninth and tenth Ebola outbreaks in DRC both started in 2018. The Équateur outbreak started in May 2018 when the DRC Ministry of Health (MOH) confirmed two EVD cases in the Bikoro health zone in the north-west of the country. Within a month, vaccination was initiated according to SAGE recommendations with delivery of the investigational vaccine,

rVSV-ZEBOV-GP, to EVD case contacts and contacts of contacts and frontline workers. This outbreak lasted 3 months and was confined to the Équateur Province. It infected 54 people and claimed 33 lives, resulting in a case fatality rate of 61%.[8] On 24 July 2018, the Équateur outbreak was declared over by the DRC MOH.

On 28 July 2018, the Provincial Health Division in North Kivu alerted the DRC MOH to a cluster of suspected EVD cases on the opposite side of the country in the north-east region. The first EVD cases were confirmed by INRB and the tenth outbreak was declared on 1 August 2018. Within 3 days, more than 76 confirmed, probable and suspected cases were reported, including HCP, spanning different health zones across North Kivu and Ituri Provinces. The MOH and INRB, with the support of the WHO and international partners, set up coordination mechanisms as well as Ebola treatment centres (ETC) and transit centres to screen for suspected EVD cases and to care for confirmed EVD cases.

On 24 November 2018, a randomized control trial began to assess several candidate Ebola treatments in patients in North Kivu. In addition, confirmed EVD patients in ETCs are offered investigational therapies and supportive care under compassionate use protocols.[9] EVD testing facilities, including field and mobile laboratories, have been set up to provide near-patient EVD diagnosis using automated polymerase chain reaction (PCR) assays. In addition, whole genome virus sequencing capacity has been set up to enable transmission chain analysis.

Despite early signs of success in outbreak control around the original epicentre near Beni, the North Kivu epidemic has continued for more than nine months with moderate intensity and is still ongoing. Virus transmission in many outbreak areas has stopped and half of all affected health zones reported no new confirmed cases in the 3 weeks before 3 March 2019. However, EVD cases continue to emerge in previously unaffected areas and health care facilities. As of 9 September 2019, 3084 total cases of EVD (2973 confirmed and 111 probable) have been reported, resulting in 2071 deaths, with an overall case fatality rate of 67%.

On 17 July 2019, the Director-General of the World Health Organization (WHO) declared the current Ebola outbreak in DRC a Public Health Emergency of International Concern under the International Health Regulations.

Rationale for the proposed UMURINZI Ebola Vaccination Campaign

More than one year after the beginning of the ongoing outbreak of EVD in DRC, new cases are continuing to emerge. The recent identification of cases of Ebola in the DRC cities of Goma and Bukavu, which share borders with Rwanda, is of particular concern for Rwanda when one considers that Rwanda has one of the highest population densities in Africa (approximately 14 times that of the DRC). This situation has prompted the Government of Rwanda to take control measures for preventing the spread of EVD into the country.

Currently, no vaccine has been licensed for the prevention of EVD by the European Medicines Agency (EMA), the US Food and Drug Administration (FDA) or the Medicines and Healthcare Products Regulatory Agency (MHRA). Several vaccine candidates have applied to the WHO for Emergency Use Assessment and Listing (EUAL), including the Ad26.ZEBOV, MVA-BN-Filo vaccine regimen as well as the rVSV-ZEBOV-GP (or rVSVΔG-ZEBOV-GP) vaccine. During the 2013-16 Ebola outbreak in Guinea, a Phase 3 cluster-randomized ring-vaccination trial using single-dose rVSV-ZEBOV-GP investigational vaccine reported 100% efficacy in protection against EVD.[10, 11] In 2016, the WHO Strategic Advisory Group of Experts (SAGE) on Immunization recommended the rapid deployment of rVSV-ZEBOV-GP in case of an EVD outbreak under an Expanded Access (compassionate use) protocol, with informed consent and Good Clinical Practice (GCP) compliance.[12]

A WHO evaluation of potential vaccine candidates has recently been completed that has evaluated the suitability of Ebola vaccines for clinical studies. Data were reviewed on two vaccines, Ad26.ZEBOV, MVA-BN-Filo and the CanSino-Beijing Institute of Biotechnology (Ad5-EBOV) vaccine. The evaluation examined immunogenicity and safety data from

preclinical and clinical studies, seroconversion, antibody persistence, vaccine stability data and vaccine availability. The Ad26.ZEBOV, MVA-BN-Filo scored higher in several areas, including non-human primate (NHP) studies, study populations, safety follow-up and stability at different temperatures. The safety and immunogenicity profiles to date and the high number of vaccine doses available (>1 million) led the Rwandan Ministry of Health selecting this vaccine for this large-scale population-based prevention program

In April 2019, WHO Strategic Advisory Group of Experts on immunization revised their vaccination strategy recommendations. In that revision, it was recommended to offer a vaccine other than rVSV-ZEBOV-GP to those at some risk of Ebola. Following SAGE (WHO) review of data generated by Ebola vaccine manufacturers on two candidate vaccines: the adenovirus 26 vectored glycoprotein / MVA-BN (Ad26.ZEBOV/ MVA-BN) vaccine developed by Johnson & Johnson, and the CanSino-Beijing Institute of Biotechnology (Ad5-EBOV) vaccine, SAGE has recommended that lower risk populations could be vaccinated with the J&J vaccine.

In July 2019, the statement on the meeting of the International Health Regulations (2005) Emergency Committee for Ebola virus disease in DRC recommended at risk-countries to “put in place approvals for investigational medicines and vaccines as an immediate priority for preparedness”. Following this recommendation, and after reviewing the application license submitted by Janssen, on 27 September 2019, Rwanda FDA granted conditional approval of the Ad26.ZEBOV and MVA-BN-Filo Ebola vaccine under exceptional emergency circumstances as part of an initiative to protect Rwandan citizens against Ebola.

Ad26.ZEBOV and MVA-BN-Filo Vaccines

Ad26.ZEBOV is a monovalent, replication-incompetent Ad26-based vector that expresses the full-length GP of Ebola virus (EBOV, formerly known as Zaire ebolavirus) Mayinga strain. It is produced in the human cell line PER.C6®.

The Ad26.ZEBOV vaccine will be supplied at a concentration of $1x10^{11}$ vp/mL in 2mL single-use glass vials as a frozen liquid to be thawed before use. Each vial contains an extractable volume of 0.5mL.

The vaccine is manufactured by IDT Biologika GmbH for Janssen Vaccines & Prevention B.V., The Netherlands.

MVA-BN®-Filo, is a recombinant multivalent Modified Vaccinia Ankara (MVA)-vectored vaccine. It expresses the EBOV GP, the Sudan virus (SUDV) GP, the Marburg virus (MARV) Musoke strain GP, and the Tai Forest virus (TAFV, formerly known as Côte d’Ivoire ebolavirus) nucleoprotein (NP).

MVA-BN-Filo is strongly attenuated; the vaccine is propagated in primary chicken embryo fibroblast (CEF) cells and does not replicate in human cells. The MVA-BN-Filo vaccine is supplied at a concentration of 2×10^8 Inf U/mL in 2mL single-use glass vials as a frozen liquid suspension to be thawed before use. Each vial contains an extractable volume of 0.5mL.

The MVA-BN-Filo vaccine is manufactured by Bavarian Nordic A/S, Denmark, for Janssen Vaccines & Prevention B.V., The Netherlands.

Clinical Studies with Ad26.ZEBOV and MVA-BN-Filo

The safety, reactogenicity, and immunogenicity of the Ad26.ZEBOV and MVA-BN-Filo vaccines have been/are being evaluated in a number of completed and ongoing clinical studies in adults and children ≥1 year of age. The Janssen’s current clinical development plan contains 4 completed Phase 1 studies (EBL1001, EBL1002, EBL1003, EBL1004), 1 completed Phase 2 study (EBL2001), 2 completed Phase 3 studies (EBL3002 and 3003) and several ongoing Phase 2/3 studies that have completed dosing and are in the follow-up phase (EBL2002, EBL2003, EBL3001 and EBL4001).

As of December 2018, an estimated 6,500 participants (adults, children, HIV+ adults) have received at least the first vaccination (Ad26.ZEBOV, MVA-BN-Filo, or placebo/active

comparator).

Phase 1/2/3 Studies in adults

Unblinded safety data from 2,390 adults from studies (EBL1001, EBL1002, EBL1003, EBL1004, EBL2001, EBL2002, EBL3001, EBL3002, EBL3003, and FLV1001), including 1,814 healthy and 118 HIV+ adults dosed with Ad26.ZEBOV, MVA-BN-Filo [N=1,932], and 434 healthy and 24 HIV+ adults dosed with control [placebo or active control, N=458]) is summarized here. This analysis included only adults who were enrolled to receive any of the Ad26.ZEBOV, MVA-BN-Filo regimens at the clinical dose level, in the right sequence and when the interval between the doses was at least 28 days. Excluded from this safety analysis were children, participants who were randomized to receive Ad26.ZEBOV only (Group 4 in study EBL2001, the Ad26.ZEBOV shedding substudy), homologous regimens, MVA-BN-Filo followed by Ad26.ZEBOV regimens, or regimens that included doses other than the proposed clinical doses. Study FLV1001 was a Phase 1 study of the multivalent Ad26.Filo vaccine, containing a control arm vaccinated with the Ad26.ZEBOV, MVA-BN-Filo 0, 56-day regimen. The safety data from this arm were included in the safety pooling.

Adverse events occurring before the first vaccination are not shown. There was also 1 subject in EBL3001 who was not randomized but vaccinated and therefore excluded from the current unblinded safety dataset.

Overall, the safety profile consists of mild to moderate adverse events of short duration with no sequelae, confirming results of the Phase 1 studies. No safety signals were identified. The frequency of Grade 3 pyrexia (≥39.0˚C) was <1% following vaccination and the incidence of any febrile response was <7.5% in any group. Serious adverse events were reported in 54 participants (2.8%) vaccinated with the active vaccine regimen and in 11 participants (2.4%) vaccinated with control.

No specific safety concern was raised in the HIV+ population.

Table 1: Frequency of Solicited and Unsolicited Adverse Events in Healthy Adults – by Dose

	Adverse Events					
	Ad26.ZEBOV N=1,932		MVA-BN-Filo N=1,672		Control [a] N=850	
	n	%	n	%	n	%
Overall solicited AEs	1,449	75.0	1,075	64.3	467	54.9
Overall solicited local AEs	1,004	52.0	830	49.6	181	21.3
Overall solicited systemic	1,294	67.0	750	44.9	381	44.8
Most frequent local solicited AE	928	48.0	770	46.1	150	17.6
Most frequent systemic solicited AE	900	46.6	505	30.2	219	25.8
Any pyrexia (defined as	143	7.4	64	3.8	29	3.4
Any solicited Grade 3	85	4.4	27	1.6	15	1.8
Any solicited local Grade 3	18	0.8	11	0.7	0	0
Any solicited systemic Grade	81	4.2	24	1.4	15	1.8
Grade 3 pyrexia (defined as	17	0.9	10	0.6	6	0.7
Overall unsolicited AEs	729	37.7	564	33.7	323	38.0

N: Number of doses of Ad26.ZEBOV, MVA-BN-Filo or placebo from studies EBL1001, EBL1002, EBL1003, EBL1004, EBL2001, EBL2002, EBL3001, EBL3002, EBL3003, and FLV1001 from regimens where Ad26.ZEBOV at the clinical dose was administered as the first dose followed by MVA-BN-Filo as the second dose when at least 28 days had elapsed between the first and second doses. Includes HIV+ and healthy adults.

[a] Placebo or active control (MenACWY in study EBL3001).

The immunogenicity of the Ad26.ZEBOV, MVA-BN-Filo vaccine regimens was also confirmed in Phase 2 and 3 studies. Focusing on the 0, 56 day schedule across the different Phase 2 and 3 studies, the observed geometric mean concentrations (GMCs) measured by EBOV GP FANG ELISA 21 days post-dose-2 were between 3,810 and 11,790 EU/mL in healthy participants. In total, 98% to 100% of participants responded to the vaccine regimen (vaccine response is defined as demonstrating at least a 2.5-fold increase in binding antibody concentration over baseline value). Some geographic differences in immune responses were observed with GMC values ranging between 10,131 and 11,790 EU/mL for EU and USA studies compared to ranging between 3,810 and 7,501 EU/mL for African studies. Available long-term follow-up data (up to 2 years) indicates that antibody responses persist in all studies, but the GMCs in the African studies tended to be lower (above 1,000 EU/mL in the EU versus 259-381 EU/mL in Africa).

Because the 2013-16 Ebola outbreak subsided before clinical efficacy data could be generated, it was agreed with regulators (EMA and FDA) to infer clinical benefit by immunobridging, i.e. assessing human immunogenicity against a model that describes the relationship between immunogenicity and survival in NHPs. It is important to keep in mind that due to differences in susceptibility between NHP and humans, the NHP model is considered to be more stringent than human EVD. The stringent nature of the NHP model will ensure that immunobridging provides a conservative estimate of the clinical benefit in humans.

A pre-planned interim analysis on pooled data from 5 pivotal Phase 2 and 3 studies (VAC52150EBL2001, EBL2002, EBL3001, EBL3002, and EBL3003) conducted in USA, EU and Africa was performed in October 2018. This analysis evaluated the vaccine regimen in healthy adults (aged 18 to 50 years, vaccinated with Ad26.ZEBOV, MVA-BN-Filo 2-dose

regimen with a 0, 56 day interval). Evaluation was done by calculating the mean predicted survival probability and its 95% confidence interval (CI).

Based on the pooled data from 764 healthy adults, the point estimate of the mean predicted survival probability is 53.4% and the lower limit of the 95% CI is 36.7%, well above the pre-specified success criterion of 20% that was agreed with regulators as a basis for licensure. The outcome of this interim immunobridging analysis therefore strongly supports the clinical benefit of the vaccination.

Phase 2/3 Studies in children

Unblinded safety information on a total of 649 children (253 adolescents aged 12-17 years, 252 children aged 4-11 years and 144 toddlers aged 1-3 years) who received the active vaccine regimen in 2 clinical studies (EBL2002, EBL3001) is summarized here. Overall, the safety profile consisted of mild to moderate adverse events of short duration in all pediatric age groups. Grade 3 Fever (≥39°C) within 7 days following vaccination was rare (<1.5%). SAEs were reported in 12 children (1.8%) in the active group and 3 children (1.6%) in the control group. All SAES were infections except for 1 anemia associated with malaria in the active group and 1 second degree burn in the control group. No SUSARs related to the Ad26.ZEBOV or MVA-BN-Filo vaccines were reported and no safety signals were identified in children 1-17 years of age.

As for adults, the Ad26.ZEBOV, MVA-BN-Filo vaccine regimen is highly immunogenic in children.

Vaccination during pregnancy

Pregnancy was an exclusion criterion for the past clinical trials in this program. A mandatory pregnancy test was performed before each vaccination and a commitment to use adequate contraception was requested from all females of childbearing potential. Inadvertent pregnancies occurring during clinical trials with the Ebola candidate vaccines Ad26.ZEBOV, Ad26.Filo, and MVA-BN-Filo have been followed to term.

The most recent aggregate review of pregnancy exposure data was performed in August 2019. This analysis of the current experience with pregnancies after exposure to the Ebola candidate vaccines (Ad26.ZEBOV, Ad26.Filo, MVA-BN-Filo) in female study participants or partners of male study participants did not reveal a safety concern.

Serious complications/SAEs during pregnancy were reported in 24 out of a total of 79 pregnancies, i.e. in 20 women study participants and in 4 women partners of male study participants, based upon the most recent aggregate review of pregnancy exposure data within Janssen's Ebola vaccine program that was performed for the period up to 12 August 2019. None of these serious complications/SAEs were considered causally associated with the study vaccines by Investigators and/or the Company. No apparent concerning pattern of AEs is emerging from this review. No congenital malformations were reported to date to the Company in fetuses or newborns. Spontaneous abortion was the most commonly observed SAE (11 out of 79 pregnancies) with an incidence of 13.9%, which is within the range of expected spontaneous abortion rates during the first trimester of gestation even when considering that spontaneous abortion incidences vary significantly depending of geographical areas and individual risk factors (e.g. age, previous abortions).

Review of safety database for SUSARs considered related to Ad26.ZEBOV or MVA BN-Filo

A review of the available safety database, including ongoing blinded studies with cut-off date of 12 December 2018 (approximately 6500 participants having received at least one vaccination (Ad26.ZEBOV, MVA-BN-Filo, or placebo/active comparator)) was also performed. Two suspected unexpected serious adverse reactions (SUSARs) have been reported in the program. Small fiber neuropathy has been reported in study EBL2001 following vaccination with Ad26.ZEBOV and generalized pruritus was reported following vaccination with

MVA-BN-Filo in study EBL2004 (PREVAC, study is still blinded and ongoing). In addition, one subject in study EBL2001 experienced a serious and very rare condition called “Miller Fisher syndrome” about 1 week after suffering from a respiratory tract infection and about 1 month after dose 2 vaccination with MVA-BN-Filo. After extensive investigation, the event was considered not to be related to study vaccine and most likely related to the prior upper respiratory tract infection by the investigator.

There are no adverse drug reactions or events of special concern listed in the Investigator’s Brochures.[13, 14]

OBJECTIVE

The objective of the UMURINZI Ebola Vaccination Campaign is to provide the Ad.26ZEBOV, MVA-BN-Filo Ebola vaccine regiment to at least 193,000 residents of Rwanda living in the vicinity of an Ebola outbreak.

IMPLEMENTATION OF THE VACCINATION CAMPAIGN

Population for Vaccination

The target population of the UMURINZI Ebola Vaccination Campaign will consist of adults, adolescents, and children aged 2 years or above who reside in the selected communities for this population-level intervention and who, at the time of vaccination with dose 1, are planning to remain in the selected area(s) for at least 2 months. At the time of vaccination, individuals who have a known allergy or history of anaphylaxis or other serious adverse reactions to vaccines or vaccine products, including known allergy to egg, egg products, and gentamycin, will be excluded from receiving the Ad26.ZEBOV, MVA-BN-Filo vaccine regimen. Pregnant women will not be vaccinated as part of this campaign. Breastfeeding women may be included.

Currently, the population deemed to be at risk for EVD consists of people residing in Rwanda who frequently cross the Rwanda / DRC border, potential first responders who have not previously been vaccinated against EVD, as well as people who reside in high-risk border-proximate areas of the Rubavu, Rutsiro, Karongi, Rusizi, and Nyamasheke districts in the Western Province.

In the first phase of this vaccination campaign, we will select 14 vaccination centers in the Rubavu and Rusizi districts. Additional vaccination centers within these districts and other districts will be added as determined by the Ministry of Health. The table below lists the first 14 vaccination centers in the Rubavu and Rusizi districts.

Table 2: List of the first 14 vaccination centers

District	Sector	Health Center
**Rubavu**	Bugeshi	Bugeshi HC
	Busasamana	Busasamana HC
	Cyanzarwe	Busigari HC
	Rubavu	Byahi HC
	Gisenyi	Gacuba HC
	Rugerero	Gisenyi HC
	Nyamyumba	Kigufi HC
**Rusizi**	Bugarama	Islamic HC
	Nzahaha	Rwinzuki HC
	Gashonga	Mibilizi HC
	Nyakarenzo	Nyakarenzo HC
	Mururu	Rusizi HC

	Kamembe	Gihundwe HC
	Gihundwe	Mont Cyangugu HC

Community engagement and social mobilization

The success of the UMURINZI Ebola Vaccination Campaign will depend on the level of trust in the community. Direct engagement with local communities will commence as early as possible. The implementation team from Projet San Francisco/Center for Family Health Initiative, Rinda Ubuzima, Rwanda Biomedical Center, district hospitals and health centers in high-risk zones will work closely with key stakeholders including provincial, district and local authorities, immigration officers, religious and other opinion leaders, community representatives, mass media, and community health workers to conduct training and sensitization campaigns in the community. These different stakeholders will be consulted and their input integrated into our community engagement approach and operations. Community engagement will be conducted to ensure the implementation team receives feedback from the community on social harms, individual and community level risks, perceptions about the vaccine, and vaccination implementation issues.

Community engagement activities will include establishment of Community Advisory Committees (CAC) with a target of involving all key stakeholders mentioned above. We plan to have regular meetings of the CAC to ensure that the constituted CAC act as a liaison contact between the vaccination centers and the target participants in the community. The CAC will provide advice before, during and after the program implementation, ensuring a transparent and meaningful participatory process, and it is envisaged that CAC meetings will be held as frequently as appropriate (at least quarterly).

Education & Information session and Biometrics Identification

The UMURINZI Ebola Vaccination Campaign will enroll only people who provide consent to be vaccinated. The process of informing individuals coming to the health center for vaccination will include provision of a Rwanda FDA-approved factsheet for Recipients on the emergency use of Ad26.ZEBOV, MVA-BN-Filo vaccine regimen (Appendix A), a group information session conducted through video or flip chart, and a questions & answers session. Following the group information session, each individual will be received privately by a staff member of the vaccination campaign. During this individual session, people will be offered another opportunity to ask more questions to ensure that they do understand the purpose of the vaccination campaign and that they freely agree to receive the vaccine. Vaccine acceptance will be confirmed by the individual agreeing to be taken a digital photograph and biometric iris scan. Basic demographic information such as the names, date of birth, gender and contact details, national ID card number, telephone number and area of residence will be collected. Biometrics, photographs and participant-held vaccination cards will be used for individual verification at the second vaccination visit or any other subsequent visit.

Vaccination and Follow-up

Following screening for fever and a brief check of the health status, a urine pregnancy test will be performed on all women of childbearing potential. Eligible individuals will receive an IM injection of Ad26.ZEBOV ($5x10^{10}$ viral particles (vp)) as the first dose of the vaccine regimen. After vaccination, people will stay in observation for at least 15 minutes. In the education/information sessions before vaccination, the participants will be informed about expected side effects of the vaccine. Vaccinated individuals will be instructed to contact the medical team for any adverse event that causes them concern, in particular if it led to urgent medical consultation or hospitalization in the next 8 weeks following the vaccination. Women of childbearing potential will also be reminded to contact the medical team if they suspect that they have become pregnant.

An appointment for the second dose will be given in approximately 8 weeks after receiving the first dose. A mobile messaging platform will be used to remind people of the second vaccination dose at least 2 weeks before the next appointment.

The second vaccine dose, MVA-BN-Filo ($1x10^8$ infectious units (Inf U)) will be administered approximately 8 weeks after the first dose. Female participants of childbearing potential will be offered a urine pregnancy test before they receive the second vaccine dose. The second vaccine injection will not be offered if the urine pregnant test is positive. Vaccinated individuals will again be given instructions to contact the medical team for any adverse event that causes them concern or if they become pregnant.

Table 3: Schedule of Activities

Procedure	Pre-Vaccination	Dose 1	~ 8 weeks after dose 1
Community Engagement	X	X	X
Information & Education		X	X
Digital picture		X	
Iris scanning		X	X
Vaccination		X	X
SAE monitoring		X	X
Pregnancy monitoring		X	X

Handling storage and dispensation of the vaccines

The Ad26.ZEBOV and MVA-BN-Filo vaccines will be shipped from Belgium to Rwanda under the responsibility of Janssen. After the vaccines have arrived in Kigali, responsibility for the vaccines will be transferred from Janssen to MOH.

The vaccines will be shipped at -20°C to the central warehouse of the Vaccine Preventable Disease Program (VPDP) Unit of RBC in Kigali, where it will be stored at -20°C, in a secured location, with no access for unauthorized personnel. Vaccines will be transported by vehicle from the central warehouse to health centers/district hospitals in frozen state in cold boxes equipped with ice packs at -20^0C (range -15 to -25^0C) and continuous temperature data loggers. At health centers, vaccine will be stored in $2\text{-}8^0$C refrigerators equipped with continuous temperature data loggers. At district hospitals, vaccines can be stored in $-15\text{-}25^0$C freezers equipped with continuous temperature data loggers.

After thawing, the vaccine can’t be returned to frozen stage. In the event that the vaccine is exposed to temperatures outside the specified temperature ranges, the Pharmacist who has received training on the evaluation of temperature excursions will review relevant data to determine if the affected vaccine can be used or should be replaced. The affected vaccine must not be used and must be quarantined. It will be destroyed upon agreement with Janssen..

Full details on the preparation, the holding time and storage conditions from the time of suspension drawing to administration of the vaccine to the recipient will be provided in the Instructions for Use document. The Instructions for Use document specifies the maximum time that will be allowed between preparation and administration of the vaccine.

Vaccinations will be given as a 0.5mL intramuscular (IM) injection into the deltoid muscle in the upper arm:

Dose 1: Ad26.ZEBOV: $5x10^{10}$ viral particles (vp) on day 1

Dose 2: MVA-BN-Filo: $1x10^8$ infectious units (Inf U) approximately 8 weeks later

If needed, the vaccination can be administered in the thigh, especially for infants. The injection site should be free from any injury or local skin conditions. The second dose of vaccine should ideally be given in the opposite arm. Further details on the administration of the vaccine

regimen are found in the Rwanda-FDA approved factsheet on the emergency use of the Ad26.ZEBOV, MVA-BN-Filo vaccine regimen by health care professionals (Appendix B).

Who cannot receive the Ad26.ZEBOV, MVA-BN®-Filo vaccine regimen?

Pregnant women should not be vaccinated as part of this campaign. Also, anyone with anaphylactic reactions to Ad26.ZEBOV, MVA-BN®-Filo, or any of their excipients, or trace residues (chicken protein and gentamicin) must not be given the regimen. Excipients of Ad26.ZEBOV include EDTA, Ethanol,L-histidine, Polysorbate 80,Sodium chloride, Sucrose, Water for injection. Excipients of MVA-BN®-Filo include Hydrochloric acid, Sodium chloride, Trometamol (Tris-hydroxymethyl-amino methane), Water for injection.

Warnings and precautions for the use of the Ad26.ZEBOV, MVA-BN®-Filo vaccine regimen

As with all injectable vaccines, appropriate medical treatment and supervision should always be readily available in case of rare anaphylactic reactions following the administration of the vaccine. Anxiety-related reactions, including vasovagal reactions (syncope), hyperventilation or stress-related reactions may occur in association with vaccination as a psychogenic response to the needle injection. It is important that precautions are in place to avoid injury from fainting. The individuals should be monitored by a healthcare professional for 15 minutes after vaccination.

Immunization should be postponed in individuals suffering from an acute severe febrile illness or acute infection. The presence of a minor infection and/or low-grade fever should not result in the delay of vaccination.

Before administering the Ad26.ZEBOV, MVA-BN®-Filo vaccine regimen, the health care staff should review the individual's medical history, particularly regarding hypersensitivity reactions to previous administration of any type of vaccine.

The vaccinated person should stay at the health center for at least 15 minutes observation.

Concomitant use with other vaccines

Any interventions required to treat a disease or condition in a person who has received the Ebola vaccine will be allowed. The safety, immunogenicity and efficacy of co-administration of the vaccine regimen regimen with other vaccines have not been evaluated, and therefore, co-administration is not recommended. People should not postpone, miss or delay receiving any recommended vaccine according to the Expanded Program on Immunization (EPI) schedules. If a post-exposure vaccine is indicated (e.g. rabies or tetanus), it must take priority over the Ebola vaccine.

If an Ebola outbreak occurs in the community whilst vaccinations are being implemented, planned vaccination activities should be clarified with the Ministry of Health.

REPORTING OF RELATED ADVERSE EVENTS AND CASES OF PREGNANCIES

From the day they receive the first dose of the vaccine to approximately one month after they receive the second dose of the vaccine, vaccinated individuals will be reminded to contact the medical team for any adverse event that has triggered urgent medical consultation or hospitalization. Women of childbearing potential will also be reminded to contact the medical team if they suspect that they have become pregnant.

If an adverse event/pregnancy is reported to the medical team by a vaccinated person, the team will follow up to assess whether the adverse event is a serious adverse event (SAE) and/or an unexpected adverse reaction related to the vaccine.

Based on ICH and EU Guidelines on Pharmacovigilance for Medicinal Products for Human Use[15]:

A serious adverse event (SAE) is any untoward medical occurrence that:

- Results in death.
- Is life threatening (participant was at risk of death at the time of the event). It does not refer to an event that hypothetically might have caused death if it were more severe.
- Results in persistent or significant disability/incapacity e.g. results in a substantial disruption of the participant's ability to carry out normal functions. This definition is not intended to include events of relatively minor medical significance such as headache, nausea, vomiting, diarrhoea, injection site reactions and accidental trauma (e.g. sprained ankle).
- Requires in-patient hospitalization or prolonging of existing hospitalization. In general, hospitalization signifies that the participant has been detained (usually involving at least a 24h stay) at the hospital or emergency ward for treatment that would not have been appropriate in an outpatient setting.
- Is a congenital anomaly/birth defect in the offspring of a study participant.
- Is an important medical event that may jeopardize the participant or may require intervention to prevent one of the other outcomes listed above should be considered serious.

An adverse reaction is an untoward and unintended response related to an investigational medicinal product (in this case, vaccine).

An unexpected adverse reaction: where the nature or severity is not consistent with the applicable product information (in this case, the Rwanda FDA-approved factsheet on the emergency use of Ad26.ZEBOV, MVA-BN-Filo vaccine regimen by health care professionals (Appendix B)).

SAE Initial Reporting

As soon as the vaccination center is aware of the occurrence of an SAE in a vaccinated individual, the Site Coordinator should immediately contact the District Physician who will initiate the investigation of the SAE. The initial SAE report will be reported by the Overall Coordinator or designee to MOH and to Rwanda FDA within 24 hours, and to Janssen within 7 days. If the SAE is fatal or life threatening, MOH, Rwanda FDA and Janssen will be informed within 24 hours.

Whenever possible, SAEs will be documented in terms of a diagnosis or syndrome rather than multiple symptoms that are clearly manifestations of the same diagnosis/syndrome. Causality of the SAEs will be assessed based on the following:

Related: there is a reasonable possibility that the vaccine caused or contributed to the SAE.

Unrelated: there is no suspicion that there is a relationship between the vaccine and the SAE; there are other more likely causes and administration of the vaccine is not suspected to have contributed to the SAE.

The following is the minimum information will be required for the initial SAE report:

- Participant’s number, and age
- Description of the event

- Date of vaccination
- Reporter information
- Preliminary causality assessment
- Severity

SAE Follow-up Reporting

SAEs will be followed through to conclusion. The follow-up report will contain new, updated or corrected information and will describe whether the event has resolved or continued, if and how it was treated, and if it is related to the vaccine or not. Only SAE related to the vaccine should be reported.

Reporting cases of pregnancy

Although not considered an AE, if a vaccinated female individual becomes pregnant after having received at least one vaccine dose, it is the responsibility of the Overall Coordinator of the vaccination campaign or designee to report the pregnancy to MOH, Rwanda FDA and Janssen within 7 days. Vaccination will be discontinued and the woman followed for safety until the end of pregnancy to record birth outcomes. If possible, approximately 2–4 weeks after delivery, the baby will be examined by a physician to assess his or her health status and the results will be reported to MOH, Rwanda FDA and Janssen. Complications of pregnancy that meet criteria for a serious adverse event, should be reported as SAEs.

Table 4. SAE Reporting Timelines

Report to	Report by	Timeline
MOH	Overall Coordinator (or designee)	For all related SAEs, within 24 hours of the study investigator becoming aware.<br><br>For cases of pregnancy, within 7 days
Rwanda FDA	Overall Coordinator (or designee)	For all related SAEs, within 24 hours of the study investigator becoming aware<br><br>For cases of pregnancy, within 7 days
Janssen Vaccines & Prevention B.V.	Overall Coordinator (or designee)	For fatal or life-threatening events, within 24 hours of the investigator becoming aware<br><br>For other related SAEs and pregnancy reports, within 7 days

ETHICAL CONSIDERATIONS

The vaccine regimen has been approved by Rwanda FDA to be used under exceptional emergency circumstances as part of an initiative to protect Rwandan citizens against Ebola, and it will be implemented as a demonstrated project. Vaccination will only be done if people agree to be vaccinated. Individuals will be fully informed of the risks and requirements of the program, and will be provided with the Rwanda FDA-approved factsheet for Recipients on the emergency use of Ad26.ZEBOV, MVA-BN-Filo vaccine regimen. SAEs related to the vaccine will be reported to Rwanda FDA, MOH and Janssen.

Potential Benefits

In total, 98% to 100% of adult participants responded to the vaccine regimen (vaccine response is defined as demonstrating at least a 2.5-fold increase in binding antibody concentration over baseline value).

In the absence of efficacy data from clinical trials, vaccine effectiveness has been inferred from immunogenicity data, through the quantification of anti-EBOV GP binding antibodies, and extrapolation from animal efficacy studies in non-human primates. Data from 5 clinical studies conducted in Europe, USA and Africa and obtained from 764 adults 18-50 years of age who had received the 2-dose vaccine regimen in the 56- day interval were used in this analysis. Human immunogenicity data were assessed against a logistic regression model that describes the relationship between EBOV GP antibodies and survival in NHP. Based on this analysis, the Ad26.ZEBOV, MVA-BN-Filo vaccine regimen can be anticipated to confer protection against EBOV disease in humans.

The immune responses to the Ad26.ZEBOV, MVA-BN-Filo vaccine regimen in children 1-17 years of age were evaluated in two randomized controlled clinical trials and were equivalent or higher to the responses in adults.

In addition to the potential protection against Ebola from the vaccine, vaccinated individuals may benefit from a temperature check and symptom screening which may lead to a free health check. Management for simple medical conditions will be offered in the field where possible, with onward referral for more complex or serious medical cases. People will benefit from the local medical insurance scheme provided by the Rwanda Social Security Board to ensure that they report any SAE and receive appropriate medical management and treatment. Participants may also benefit from further information about other health conditions including signs and symptoms of EVD and how to protect themselves from inadvertent exposure and where to attend if they develop symptoms suggesting of EVD. All participants may also expect to benefit from contacts with members of the study team, referrals for medical care if needed and benefit from other interventions potentially provided by the national health care system.

Potential Risks

Like many vaccines, Ad26.ZEBOV, MVA-BN-Filo vaccination may elicit some local and systemic reactions. The safety of the vaccine regimen in adults has been assessed in 11 clinical trials. A total of 2,253 adults received the vaccine regimen.

The most common local adverse reactions (≥ 10%) reported in adults who received the vaccine regimen were pain (59%), warmth (33%), swelling (17%) and pruritus (14%) at the injection site. The most common systemic adverse reactions (≥ 10%) were fatigue (54%), headache (52%), myalgia (45%), arthralgia (31%) and chills (28%). Most adverse reactions occurred within 7 days following vaccination and were usually mild to moderate in severity and of short duration (2-3 days).

The safety of the vaccine regimen in children has been assessed in 2 randomized, controlled clinical trials. A total of 649 children received the vaccine regimen: 144 were 1 to 3 years of age, 252 were 4 to 11 years of age and 253 were 12 to 17 years of age.

The most common local adverse reaction (≥ 10%) reported in children 1 to 17 years of age who received the vaccine regimen was pain (34%) at the injection site. The most common systemic adverse reactions (≥ 10%) were decreased activity (20%), decreased appetite (19%), myalgia (15%), irritability (15%), vomiting (13%), arthralgia (12%) and pyrexia (12%). Most adverse reactions occurred within 7 days following vaccination. Most adverse reactions were usually mild to moderate in severity and of short duration (1-4 days).

Fever was reported more frequently for younger children, 1 to 3 year of age (18%) and 4 to 11 years of age (15%) compared to adolescents 12 to 17 years of age (6%) and adults (7%). The frequency of fever in younger children was similar to that observed in the control group receiving a licensed paediatric vaccine.

Risk-Benefit Statement

The benefit of vaccination to provide recipients with access to an initial supply of the Ad26.ZEBOV, MVA-BN®-Filo vaccine regimen is expected to outweigh the risks in the context of the UMURINZI project.

DATA HANDLING

Privacy of personal data

The collection and processing of personal data from participants enrolled in this vaccination program will be limited to those data that are necessary to fulfill the objectives of the program. Only staff who have received proper training on Human Subjects Protection will have access to these records. Representatives of the Ministry of Health, RNEC, Rwanda FDA and Janssen may also have access to the data. Janssen will only have access to de-identified data.

Data collection

Only data that are needed to fulfill the objectives of the program will be collected. The participant's ID number will be generated from pre-printed vaccination card and linked to biometric data collected by an iris scan device in order to confirm identity and vaccination status. This will be done at the first and second vaccination visits. All data will be uploaded to a server accessible by authorized personnel only. Ongoing information on the number of persons vaccinated will be provided on an accessible dashboard for the Ministry of Health, Janssen and other stakeholders. No personal or identifying information will be included

Data Sharing Policy

A data sharing agreement will be made between the Ministry of Health, Projet San Francisco/Center for Family Health, Janssen and other stakeholders in the intervention following the WHO Joint Statement.

Publication and Authorship Policy

The findings from this vaccination campaign may be published in peer-reviewed scientific journals and disseminated at appropriate national and international conferences. All stakeholders in the program will determine the specific topics and numbers of publications, with rights to authorship being determined by contribution to the program design, implementation, and analysis, as is specified by most major scientific journals. Preference will be given for publication in peer-reviewed, open-access journals with appropriate readership and high impact factors. Consistent with Good Publication Practices and International Committee of Medical Journal Editors (ICMJE) guidelines, the Sponsor has the right to publish such primary data and information without approval from the investigator. The investigator has the right to publish study site-specific data after the primary data are published.

If an investigator wishes to publish information from the program, a copy of the manuscript must be provided to the Ministry of Health for review at least 30 days before submission for publication or presentation. Expedited reviews will be arranged for abstracts, poster presentations, or other materials. Authorship of publications resulting from this intervention will be based on the guidelines on authorship, such as those described in the ICMJE Recommendations for the Conduct, Reporting, Editing and Publication of Scholarly Work in Medical Journals, which state that the named authors must have made a significant contribution to the conception or design of the work; or the acquisition, analysis, or interpretation of the data for the work; and drafted the work or revised it critically for important intellectual content; and given final approval of the version to be published; and agreed to be accountable for all aspects of the work in ensuring that questions related to the accuracy or integrity of any part of the work are appropriately investigated and resolved.

REFERENCES

1. Blumberg L, E.D., Bausch DG, *Viral Haemorrhagic Fevers*. Manson's Tropical Diseases. Vol. Chapter 16. 2014, Oxford: Elsevier Expert Consult.
2. Heffernan, R.T., et al., *Low seroprevalence of IgG antibodies to Ebola virus in an epidemic zone: Ogooue-Ivindo region, Northeastern Gabon, 1997.* J Infect Dis, 2005. **191**(6): p. 964-8.
3. W.H.O., *World Health Organization Ebola Response Roadmap Situation Report*, in *Ebola Situation Report*. 2014.
4. Leroy, E.M., et al., *Human Ebola outbreak resulting from direct exposure to fruit bats in Luebo, Democratic Republic of Congo, 2007.* Vector Borne Zoonotic Dis, 2009. **9**(6): p. 723-8.
5. Leroy, E.M., et al., *Fruit bats as reservoirs of Ebola virus.* Nature, 2005. **438**(7068): p. 575-6.
6. Deen, G.F., et al., *Ebola RNA Persistence in Semen of Ebola Virus Disease Survivors - Final Report.* N Engl J Med, 2017. **377**(15): p. 1428-1437.
7. Bah, E.I., et al., *Clinical presentation of patients with Ebola virus disease in Conakry, Guinea.* N Engl J Med, 2015. **372**(1): p. 40-7.
8. W.H.O., *World Health Organization Ebola Virus Disease DRC External Situation Report 17*, in *Équateur*. 2018, World Health Organization.
9. W.H.O., *World Health Organization Consultation on Monitored Emergency Use of Unregistered and Investigational Interventions(MEURI) for Ebola Virus Disease (EVD)*, in *Notes for the record*. 2018, World Health Organization.
10. Friedrich, B.M., et al., *Potential vaccines and post-exposure treatments for filovirus infections.* Viruses, 2012. **4**(9): p. 1619-50.
11. Henao-Restrepo, A.M., et al., *Efficacy and effectiveness of an rVSV-vectored vaccine in preventing Ebola virus disease: final results from the Guinea ring vaccination, open-label, cluster-randomised trial (Ebola Ça Suffit!).* The Lancet, 2017. **389**(10068): p. 505-518.
12. W.H.O., *World Health Organization SAGE Ebola Virus Disease, Meeting of the Strategic Advisory Group of Experts on Immunization, October 2018 - conclusions and recommendations*, in *WHO SAGE Recommendations*. 2018: Weekly epidemiological record. p. 676.
13. B.V., J.V.P., *Investigator's Brochure JNJ-61210474 (Ad26.ZEBOV)*, in *Prophylactic Ebola vaccine*. 2019, Janssen Vaccines & Prevention B.V.
14. B.V., J.V.P., *Investigator's Brochure JNJ-63839880 MVA-BN®-Filo vaccine (MVA-BN-Filo [MVA-mBN226B])*, in *Prophylactic Filovirus Vaccine*. 2018, Janssen Vaccines & Prevention B.V.
15. ICH, *ICH Harmonised Tripartite Guideline*, in *Clinical Safety Data Management: Definitions and Standards for Expedited Reporting E2A*, ICH, Editor. 1994, ICH Expert Writing Group.
16. ICH. *Guideline for Good Clinical Practice*. Integrated Addendum to ICH E6 (R1) 2016 9 November 2016; E6(R2):[Available from: https://www.ich.org/products/guidelines.html.
