# Supplementary material for: Contraception use and pregnancy in women receiving a 2-dose Ebola vaccine in Rwanda: A retrospective analysis of UMURINZI vaccination campaign data
Source: PLoS Med. 2025 Feb 11;22(2):e1004508. doi: 10.1371/journal.pmed.1004508 (PMC11813098; doi:10.1371/journal.pmed.1004508)
Supplement: S2 Text — (PDF) [file pmed.1004508.s008.pdf]

Ad26.ZEBOV, MVA-BN®-Filo vaccine regimen
Emergency Use Authorization Fact Sheet for Recipients

You are receiving the Ad26.ZEBOV, MVA-BN®-Filo vaccine regimen as part of a public health emergency use program because you have the potential to be exposed to the Ebola virus, which can be deadly. You do not have to get this vaccine, but taking the Ad26.ZEBOV, MVA-BN®-Filo vaccine regimen to prevent Ebola virus disease (EVD) will reduce your risk of getting sick and dying. If possible, you may want to discuss with a healthcare professional the benefits and risks described in this fact sheet, or any available alternatives.

The full course of vaccination takes approximately 2 months. If you have just received the first vaccination, public officials will announce where and when you will receive the second vaccination in this regimen.

What is Ebola virus disease (EVD)?

EVD is potentially associated with severe hemorrhagic fever that can lead to serious illness, ***including death***.

- First symptoms are tiredness, fever, weakness, dizziness and muscle aches.
- Later symptoms are bleeding under the skin, internal organs and from the mouth, eyes or ears. Some people develop severe diarrhea, shock, mental confusion, seizures, kidney failure and coma that often ends in death.

How is the Ebola virus transmitted?

The virus spreads through direct contact (such as through broken skin or mucous membranes in the eyes, nose, or mouth) with:

- Blood or body fluids (urine, saliva, sweat, feces, vomit, breast milk, and semen) of a person who is sick with or has died from EVD
- Objects (such as needles and syringes) contaminated with body fluids from a person sick with EVD or the body of a person who died from EVD
- Infected fruit bats or nonhuman primates (such as apes and monkeys)
- Semen from a man who recovered from EVD (through oral, vaginal, or anal sex). The virus can remain in certain bodily fluids (including semen) of a patient who has recovered from EVD, even if they no longer have symptoms of severe illness.

RWANDA FOOD AND DRUGS AUTHORITY • RWANDA FDA

When someone gets infected with the Ebola virus, they will not show signs or symptoms of illness right away. The Ebola virus cannot spread to others until a person develops signs or symptoms of EVD. **After a person infected with Ebola develops symptoms of illness, they can spread the Ebola virus to others.**

Additionally, the Ebola virus usually is not transmitted by food. However, in certain parts of the world, the Ebola virus may spread through the handling and consumption of bush meat (wild animals hunted for food). There is no evidence that mosquitoes or other insects can transmit the Ebola virus.

Symptoms may appear anywhere from 2 to 21 days after contact with the virus, with an average of 8 to 10 days. See a doctor immediately if you have symptoms.

Rwanda Food and Drugs Authority

Approval Date: 24 OCT 2019

Expiration Date: 24 OCT 2020

What is the Ad26.ZEBOV, MVA-BN®-Filo vaccine regimen?

The Ad26.ZEBOV, MVA-BN®-Filo vaccine regimen is indicated for active immunization of individuals ≥2 years of age for prevention of Ebola virus (*Zaire ebolavirus* species) disease.

The Rwanda Food and Drug Administration has authorized the use of the Ad26.ZEBOV, MVA-BN®-Filo vaccine regimen for this emergency situation. If you get the regimen as directed and begin to feel sick anyway, get medical care right away.

How is the Ad26.ZEBOV, MVA-BN®-Filo vaccine regimen given?

The regimen consists of vaccination with Ad26.ZEBOVfollowed by a vaccination with MVA-BN®-Filo approximately 8 weeks later. The vaccination will be administered into the muscle of your arm.

Who should receive the Ad26.ZEBOV, MVA-BN®-Filo vaccine regimen?

This regimen is intended as pre-exposure prophylaxis for all individuals deemed at risk of exposure to the Ebola Zaire virus, such as first responders, healthcare workers, individuals required to travel to a confirmed or probable Ebola outbreak area.

Who should NOT get the Ad26.ZEBOV, MVA-BN®-Filo vaccine regimen?

Any one allergic to Ad26.ZEBOV, MVA-BN®-Filo, or any of the ingredients of the vaccine regimen including potential small amounts of chicken protein or gentamicin should *not* begivenMVA-BN®-

RWANDA FOOD AND DRUGS AUTHORITY • RWANDA FDA

Filo.

Ingredients of Ad26.ZEBOV include EDTA, Ethanol, L-histidine, Polysorbate 80,Sodium chloride, Sucrose, Water for injection. Ingredients of MVA-BN®-Filo include Hydrochloric acid, Sodium chloride, Trometamol (Tris-hydroxymethyl-amino methane), Water for injection

Can the Ad26.ZEBOV, MVA-BN®-Filo vaccine regimen be used in pregnant women and nursing mothers?

There are limited data available on the use of the Ad26.ZEBOV, MVA-BN®-Filo vaccine regimen in pregnant women and it is not known whether the components of the Ad26.ZEBOV, MVA-BN®-Filo vaccine regimen are excreted in human milk. During an emergency, the benefit of preventing Ebola Virus Disease may outweigh the risk in pregnant women and nursing mothers.

What are the most common side effects of the Ad26.ZEBOV, MVA-BN®-Filo vaccine regimen?

The most common local adverse reactions (≥ 10%) reported in adults who received the vaccine regimen were pain (59%), warmth (33%), swelling (17%) and itchiness (14%) at the injection site. The most common systemic adverse reactions (≥ 10%) were fatigue (54%), headache (52%), muscle pain (45%), joint pain (31%) and chills (28%).Most adverse reactions occurred within 7 days following vaccination and were usually mild to moderate in severity and of short duration (2-3 days).

The most common local adverse reaction (≥ 10%) reported in children 1 to 17 years of age who received the vaccine regimen was pain (34%) at the injection site. The most common systemic adverse reactions (≥ 10%) were decreased activity (20%), decreased appetite (19%), muscle pain (15%), irritability (15%), vomiting (13%), joint pain (12%) and fever (12%). Most adverse reactions occurred within 7 days following vaccination. Most adverse reactions were usually mild to moderate in severity and of short duration (1-4 days).

Fever was reported more frequently for younger children, 1 to 3 year of age (18%) and 4 to 11 years of age (15%) compared to adolescents 12 to 17 years of age (6%) and adults (7%). The frequency of fever in younger children was similar to that observed in the control group receiving a licensed pediatric vaccine.

What are other possible serious side effects of the Ad26.ZEBOV, MVA-BN®-Filo vaccine

RWANDA FOOD AND DRUGS AUTHORITY - RWANDA FDA

regimen?

As with all injectable vaccines, an anaphylactic reaction can occur. Symptoms of anaphylaxis include skin rashes, itching, hives, swelling of the lips, tongue or throat, trouble breathing, dizziness, fainting, stomach pain, vomiting and diarrhea.

What is unknown about the emergency use of the Ad26.ZEBOV, MVA-BN®-Filo vaccine regimen?

It is the Food and Drug Administration assessment that the benefit of providing you with emergency access to the Ad26.ZEBOV, MVA-BN®-Filo vaccine regimen is expected to outweigh the risks.Rwanda Food and Drugs Authority has given its conditional approval to use this vaccine in an emergency response to protect Rwanda residents against Ebola disease caused by Ebola Zaire virus.

Vaccination with Ad26.ZEBOV, MVA-BN®-Filo vaccine regimen might not protect all individuals against Ebola virus disease. Vaccinated individuals should adhere to local guidelines and recommendations to prevent or treat exposure to Ebola virus.
The vaccine regimen does not prevent diseases caused by Filo viruses other than Zaire Ebola virus.

By agreeing to have my iris(es) scanned, I acknowledge that I have understood and agree to what is described above and I agree to being vaccinated in this emergency setting with the Ad26.ZEBOV, MVA-BN®-filo vaccine regimen

How do I report Adverse Events or Medication Errors?

Report vaccine adverse effects or administration errors to your health care facility.

RWANDA FOOD AND DRUGS AUTHORITY • RWANDA FDA
